# An enumeration of Philippine vascular plants in Samar Island Natural Park: floristic evidence for outstanding universal value

**DOI:** 10.3897/BDJ.14.e182643

**Published:** 2026-07-21

**Authors:** Eiana Joshier A Odulio, Jorge Anton D Ordas, Yen Ting Chen, Niña Kathryn G Alfeche, Manimar Druselle V Operio, Sarah Grace S Zamudio, Roanne B Romeroso, Propa Joy S Venturina, Rudolph Valentino A Docot, Alyssa Marie A Lola, Jay Edneil C Olivar, Marjorie delos Angeles, Antonio Felipe T. Arbias, Danilo N Tandang, Grecebio Jonathan D Alejandro, Cecilia B Moran

**Affiliations:** 1 The Graduate School, University of Santo Tomas, Manila, Philippines The Graduate School, University of Santo Tomas Manila Philippines; 2 Department of Biological Sciences, College of Science, University of Santo Tomas, Manila, Philippines Department of Biological Sciences, College of Science, University of Santo Tomas Manila Philippines; 3 Biology Department, College of Medical Technology, Trinity University of Asia, Quezon City, Philippines Biology Department, College of Medical Technology, Trinity University of Asia Quezon City Philippines; 4 Department of Biology, College of Science, De La Salle University, Manila, Philippines Department of Biology, College of Science, De La Salle University Manila Philippines; 5 Department of Biological Sciences, College of Science, University of Eastern Philippines, Catarman, Northern Samar, Philippines Department of Biological Sciences, College of Science, University of Eastern Philippines Catarman, Northern Samar Philippines; 6 Department of Biological Sciences, Institute of Arts and Sciences, Far Eastern University, Manilla, Philippines Department of Biological Sciences, Institute of Arts and Sciences, Far Eastern University Manilla Philippines; 7 Deparment of Molecular Evolution and Plant Systematics and Herbarium (LZ), Institute of Biology, Leipzig University, Leipzig, Philippines Deparment of Molecular Evolution and Plant Systematics and Herbarium (LZ), Institute of Biology, Leipzig University Leipzig Philippines; 8 Institute of Biological Sciences, University of the Philippines Los Baños, Laguna, Philippines Institute of Biological Sciences, University of the Philippines Los Baños Laguna Philippines; 9 Philippine Native Plants Conservation Society, Inc., Manila, Philippines Philippine Native Plants Conservation Society, Inc. Manila Philippines; 10 Philippine National Herbarium, Botany and National Herbarium Division, National Museum of Natural History, National Museum of the Philippines, Manila, Philippines Philippine National Herbarium, Botany and National Herbarium Division, National Museum of Natural History, National Museum of the Philippines Manila Philippines; 11 Research Center for the Natural and Applied Sciences, University of Santo Tomas, Manila, Philippines Research Center for the Natural and Applied Sciences, University of Santo Tomas Manila Philippines

**Keywords:** forests over limestone, Philippine flora, plant inventory, Samar flora, tracheophyta

## Abstract

**Background:**

Samar Island Natural Park (SINP), the largest terrestrial protected area in the Philippines, encompasses the nations's most extensive karst landscape and largest remaining intact old-growth forests. While it is widely recognised for its exceptional biodiversity, comprehensive information for SINP, especially for plants, remains elusive. As a result, major taxonomic and distributional gaps persist. Nevertheless, with its immense biological value and urgent conservation needs, SINP has been nominated as a UNESCO World Heritage Site. To bolster this nomination, field surveys combined with secondary data were performed to generate a checklist of vascular flora of SINP.

**New information:**

A total of 874 morphospecies representing 124 families and 444 genera were documented in SINP. The flora is predominantly native and exhibits high endemism, including 339 Philippine endemics and 37 species restricted to Samar Island, alongside 82 newly-recorded species for the island. The taxonomic composition, dominated by Rubiaceae, Orchidaceae and other species-rich families, reflects both regional floristic patterns and SINP’s biogeographical positioning within the Sunda–Sahul Convergence Zone. Moreover, a notable portion of its flora is threatened under the IUCN and DENR-DAO listings, with the majority classified as Not Evaluated. Collectively, these findings demonstrate that SINP functions as a critical refuge for some of the Philippines’ most unique and vulnerable plant lineages.

## Introduction

Botanical research in the Philippines has progressed significantly since the early 20^th^ century, shaped initially by extensive taxonomic work and regional floristic inventories. Amongst these early contributions, Merrill's *Enumeration of the Philippine Flowering Plants* (Merrill 1926) has laid a critical foundation for understanding the archipelago's diverse flora. Yet, even with this historical groundwork, our understanding of the country's true plant diversity and its distributions is plagued by Linnaean and Wallacean shortfalls (Lomolino 2004, Possingham et al. 2007). This is made even more evident given the Philippines' status as a top priority hotspot with exceptionally high plant endemism (Myers et al. 2000) and its recent identification as a plant diversity darkspot of global collection priority (Ondo et al. 2024). Such data and knowledge gaps are especially alarming in light of ongoing deforestation and the compounding effects of invasive alien species and climate change, all of which accelerate habitat loss and render many narrow, endemic and evolutionarily distinct lineages vulnerable to extinction. This amalgamation of high biodiversity value and insufficient documentation emphasises the need for further botanical explorations, systematic collections and the establishment of vital ecological baselines to identify centres of endemism, assess species rarity and threat status and refine conservation priorities. Such efforts are essential for providing information for national management decisions and supporting international recognition of areas with outstanding biological importance. Within this broader context, the biologically rich landscapes of Samar Island represent high-priority sites for comprehensive assessments.

Samar Island is the third-largest landmass in the Philippines, with 603 islands and a total area of 13,428.8 km^2^ and is globally recognised for its extensive karst landscape (Restificar et al. 2006) that accommodates the largest remaining contiguous tract of old-growth forest left in the country (Patindol 2016). As an important biogeographic zone, it hosts some of the most remarkable assemblages of endemic flora and fauna throughout the archipelago and is home to various threatened species (Madulid 2000). More recently, the herpetological expedition of Diesmos et al. (2026) provided a significant update of this Island's terrestrial biodiversity, highlighting the need for continuous assessments in this hotspot. Due to its astounding biological value, which requires conservation and management, the Samar Island Natural Park (SINP) was established under Proclamation No. 442 of the National Integrated Protected Areas System (NIPAS) in 2003, covering a total area of 330,300 hectares.

Numerous biological assessments in the SINP have demonstrated its rich and outstanding biodiversity, especially true to its highly endemic flora. Two decades ago, Madulid (2000) documented at least 400 endemic species of angiosperms belonging to approximately 200 genera. With new developments that deepen our taxonomic understanding of the nature of our biodiversity, in addition to numerous floristic expeditions conducted in the past decade to address Wallacean shortfalls (e.g. Quimio (2016), Ordas et al. (2019), Obeña et al. (2021), Villanueva et al. (2021a), Villanueva et al. (2021b)), it is expected that these values will grow through the years. More than twenty species were described in the past decade alone (e.g. Banag et al. (2015),Ordas et al. (2017), Adorador (2019), Adorador and Fernando (2019), Meneses and Cootes (2019), Tandang et al. (2019), Adorador et al. (2020), Chavez et al. (2020), Adorador et al. (2021), Rubite et al. (2021), delos Angeles et al. (2022a), delos Angeles et al. (2022b), Ordas et al. (2022), Tandang et al. (2022), delos Angeles et al. (2024), Fontarum-Bulawin et al. (2024), Docot et al. (2026)), representing good progress towards bridging the Linnaean shortfalls, with more undescribed species awaiting discovery in its large, yet unexplored forested areas of the protected landscape. However, SINP continues to face rampant anthropogenic activities, including logging, land conversion and pollution, leading to biodiversity declines and the extinction of narrow-endemic species. Therefore, collecting specimens and conducting botanical surveys are crucial for formulating specific conservation and management policies to prevent further biodiversity loss.

Despite its status as a biodiversity hotspot, the vascular flora of SINP remains poorly documented overall. Therefore, this study aims to establish a definitive floristic baseline by synthesising field surveys, published literature and database records. This inventory is significant as it provides strong empirical evidence of the Park's "outstanding universal value", offering an important dataset required for its nomination as a UNESCO World Heritage site.

## Materials and methods

### Study site

Samar Island Natural Park (11°49'49"N, 125°10'00"E) is situated in the centre of Samar Island, the easternmost island landmass in the central Philippines. It faces the Philippine Sea to the east and the Leyte Gulf to the south (Lancion and Guzman 1995), with the entire Island experiencing humid conditions year-round (Kintanar 1984). Due to Samar's geographical location, it suffers greatly from tropical cyclones. SINP specifically is characterised by Type II and IV climate, with the former having heavy rainfall with no dry season from December to January, whereas the latter bears an even distribution of rainfall throughout the entire year (Madulid 2000).

**Figure 1.**
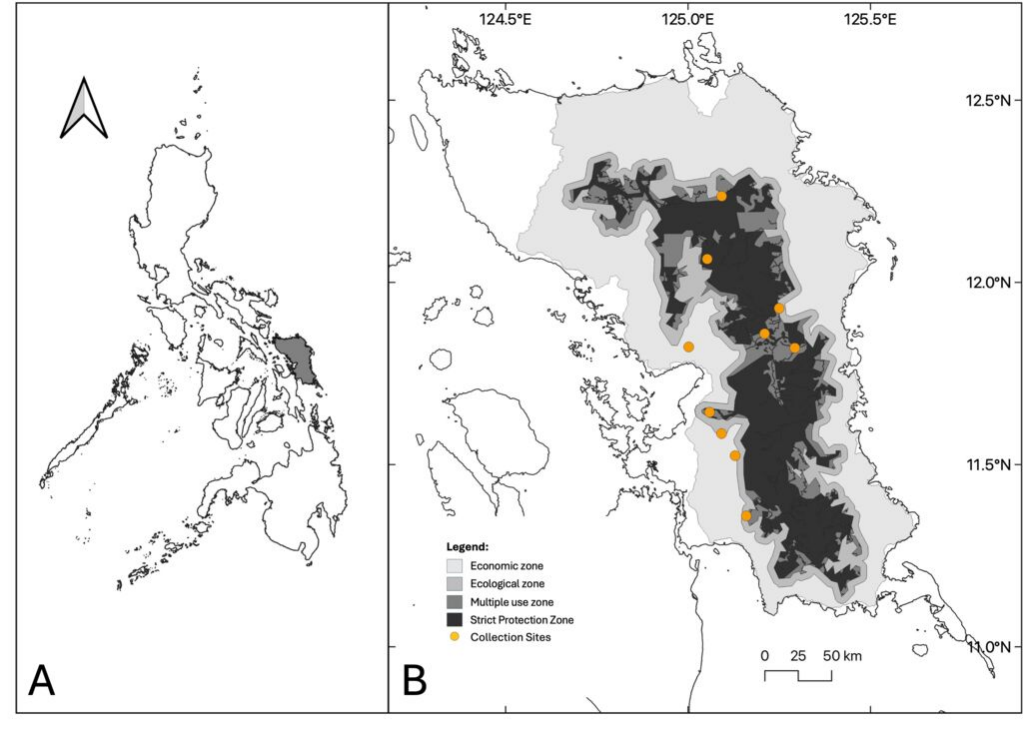
Map of sampling sites in Samar Island Natural Park (SINP). **A** Philippine map; **B** Island of Samar showing the Protected area and study sites.

**Figure 2.**
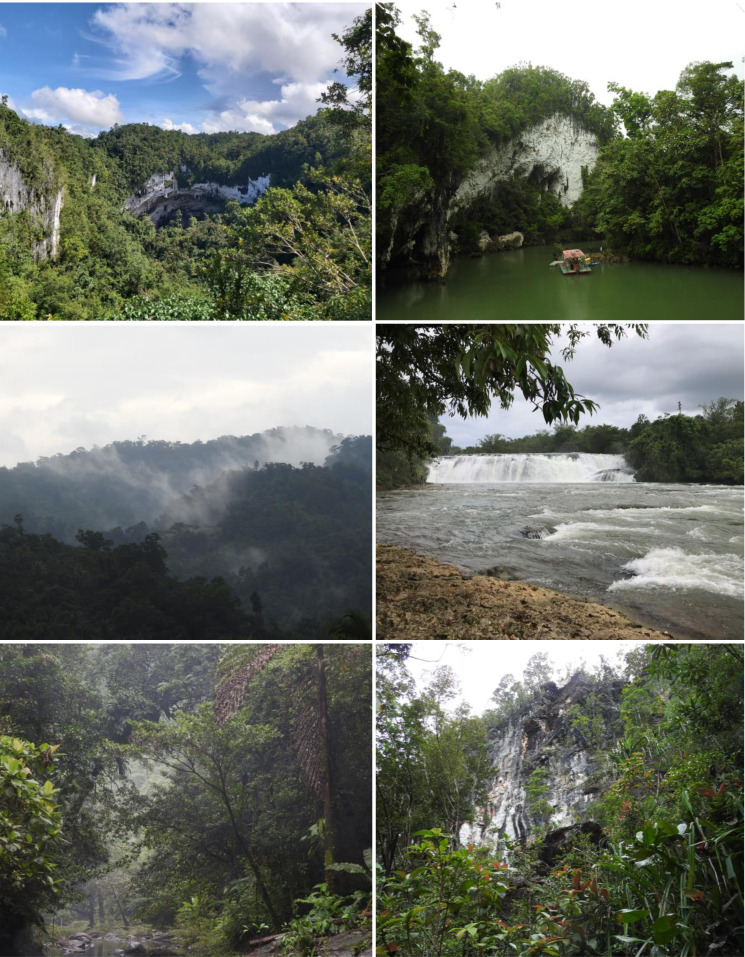
Vegetation and limestone karst formations in SINP. Photographs by NALEC, YLDP and AFTA.

Fieldwork for the Samar Island Natural Park (SINP) was executed across 11 strategically selected sites, in coordination with the Department of Environment and Natural Resources (DENR) and local government units (LGUs), spanning the three provinces of Northern Samar (San Isidro and Las Navas), Eastern Samar (Brgy. Boco, Can-Avid; San Rafael, Taft) and Samar (Mt. Huraw, San Jose de Buan; Ulot Watershed and Brgy. Tenani, Paranas; Brgy. Canlobo, Pinabacdao; Lulugayan Falls and Langun-Gobingob Cave, Calbiga; Sohoton, Basey; and Brgy. Calantawan, Motiong) (Fig. 1). To ensure the checklist was representative of the Park's complex landscape, sampling sites encompassed a broad spectrum of habitats, including old-growth primary forests, lowland evergreen forests and lowland secondary forests. Notably, the survey included significant karst formations and forests over limestone (Fig. 2), several of which were biologically unexplored prior to this study, thereby allowing documentation of specialised flora across diverse ecological niches.

### Field collection and specimen identification

Two expeditions were performed, with the first fieldwork from October to November 2021 and the second fieldwork in July 2022. Access to SINP's central parts were limited due to the: 1) lockdowns in the Covid-19 pandemic; 2) heightened insurgencies and 3) very dense primary vegetation without any established trails. During our surveys, opportunistic collections were performed, with at least three to five branches of the plant bearing reproductive parts being gathered. Their ecology, notable morphological characteristics and GPS coordinates were noted and photographs were taken in their natural habitat. The reproductive parts, such as flowers and fruits, were preserved in 70% ethanol and the herbarium specimens were deposited in the University of Santo Tomas Herbarium (USTH).

Collected specimens were mainly identified utilising protologues, revisions and taxonomic keys. The collections were also compared to specimens deposited in USTH and to type images from virtual herbaria and from available online resources, such as Co's Digital Flora of the Philippines (Pelser et al. 2011, onwards), Plants of the World Online (POWO 2025) and Global Plants on JSTOR.

## Checklists

### A species list of vascular plants in Samar Island Natural Park

#### Tracheophyta

C07CE42C-616C-58FB-B88D-8A216AF3D30D

#### Lycopodiopsida, Polypodiopsida, Cycadopsida, Gnetopsida, Pinopsida, Liliopsida and Magnoliopsida

AA006B65-541D-5966-A482-8D7A5010AFEA

##### Notes

A species list (Table 1) was generated by integrating collection data, published literature and Global Biodiversity Information Facility (GBIF) records. GBIF occurrences were subsequently filtered to species exclusively within the SINP protected area boundary. Information about the endemism of the species to the Philippines and specifically to Samar Island is included. The conservation status of each species is based on DENR (2026) Administrative Order No. 2026-20 (DAO 2026-20) and the IUCN Red List of Threatened Species (IUCN 2025). The records generated during the fieldwork campaign have been successfully uploaded and integrated into the GBIF database as occurrence dataset, accessible through this link: https://doi.org/10.15468/9vcfsu (Odulio and Alfeche 2025).

## Analysis

**Figure 3.**
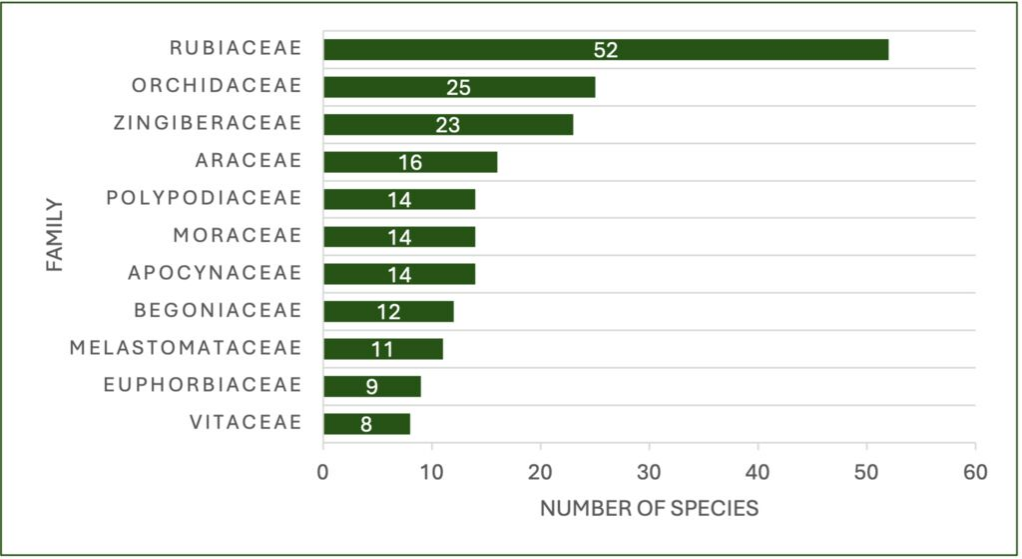
The top species-rich families, based on the fieldwork performed in this study.

Our field surveys yielded a total of 515 collected specimens, representing 382 morphospecies, 226 genera and 84 families. Of these, 322 angiosperms, 57 ferns and allies and two gymnosperms are recorded. The most species-rich families are Rubiaceae (52), Orchidaceae (25), Zingiberaceae (23) and Araceae (16) (Fig. 3), whereas all remaining families contain 14 species or fewer. In addition, 82 species represent new locality records for both SINP and Samar as a whole.

Secondary data from published literature and GBIF yielded 383 and 298 species, respectively, with 65 recurring species between the two sources. Integrating our field collections with secondary sources, the consolidated checklist records 874 vascular plant species within SINP, belonging to 444 genera and 124 families. This represents the updated floristic baseline for the entire park, effectively addressing the spatial gaps in previous literature which focused only on isolated sectors of the protected area.

**Figure 4.**
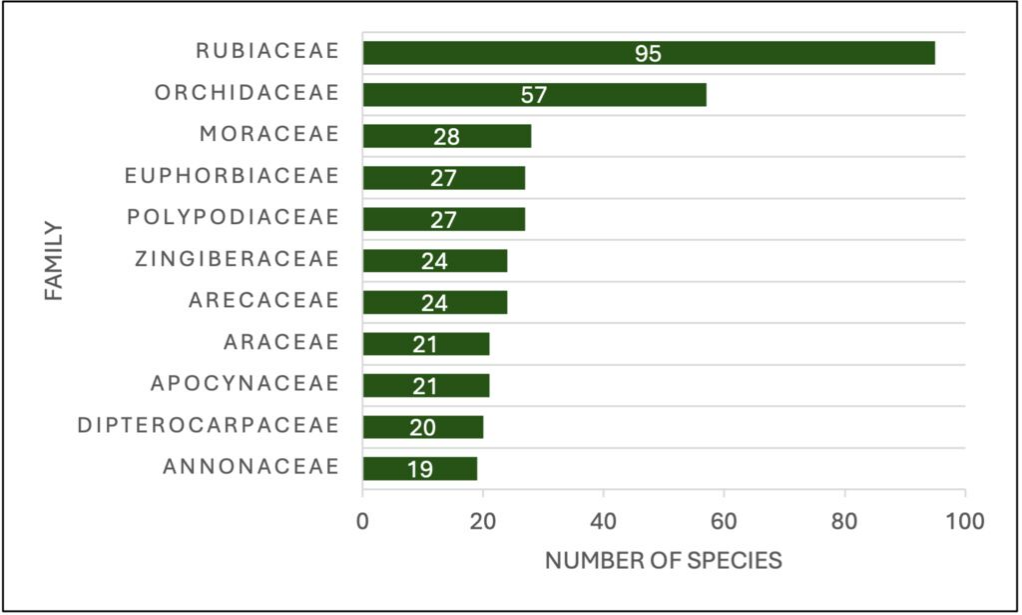
The top species-rich families in SINP, based on fieldwork collections and compiled secondary data.

**Figure 5.**
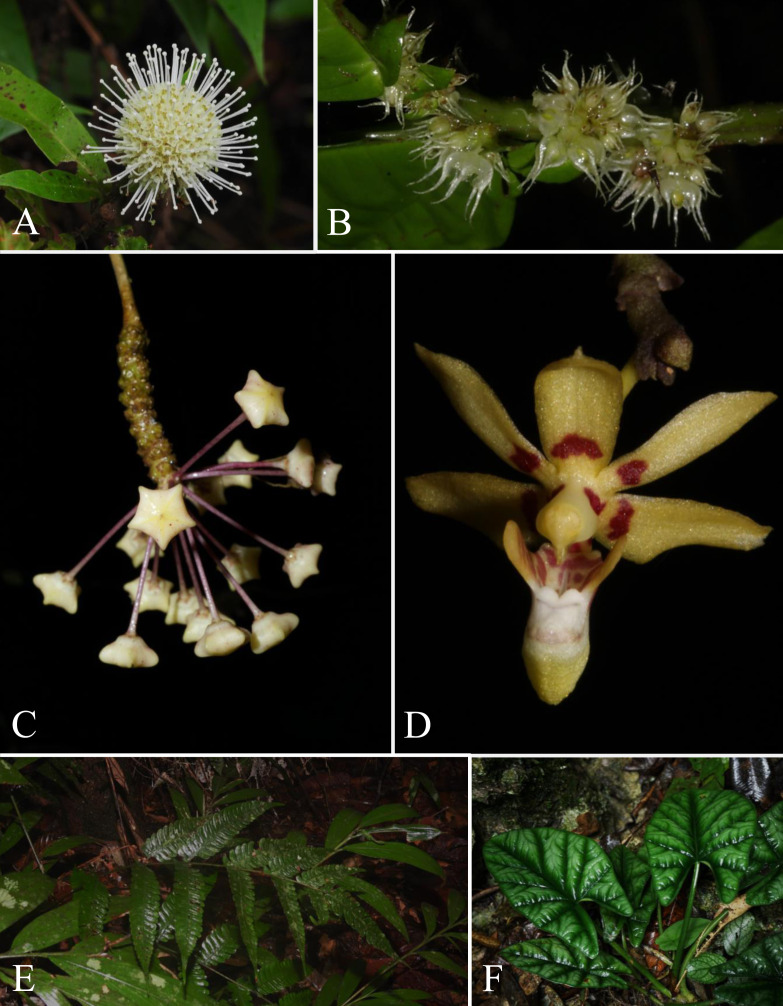
Selected Philippine endemic species occurring in SINP. **A** *Neonauclea jagorii*; **B** *Elatostema volubile*; **C** *Hoya pentaphlebia*; **D** *Pteroceras philippinense*; **E** *Pneumatopteris glabra*; **F** *Alocasia sinuata*. Photographs by NKGA, SGSZ, RVAD and AFTA.

**Figure 6.**
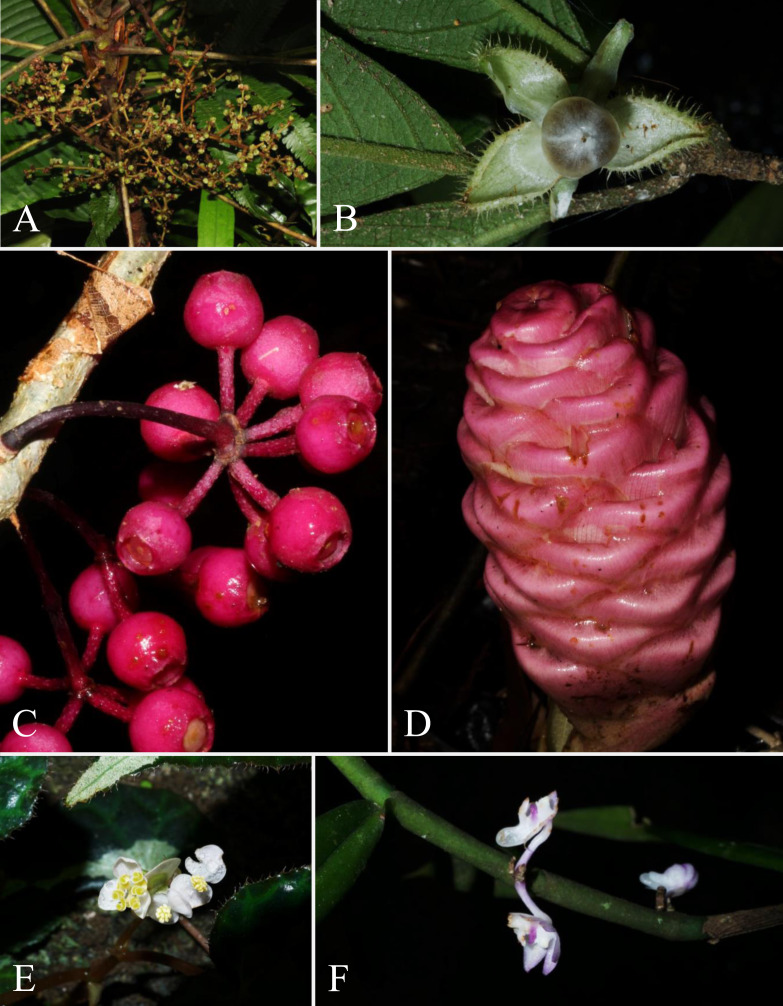
Selected Philippine endemic species occurring in SINP. **A** *Macaranga ovatifolia*; **B** *Saurauia samarensis*; **C** *Medinilla copelandii*; **D** *Zingiber subroseum*; **E** *Begonia colorata*; **F** *Trichoglottis latisepala*. Photographs by NKGA, SGSZ and RVAD.

**Figure 7.**
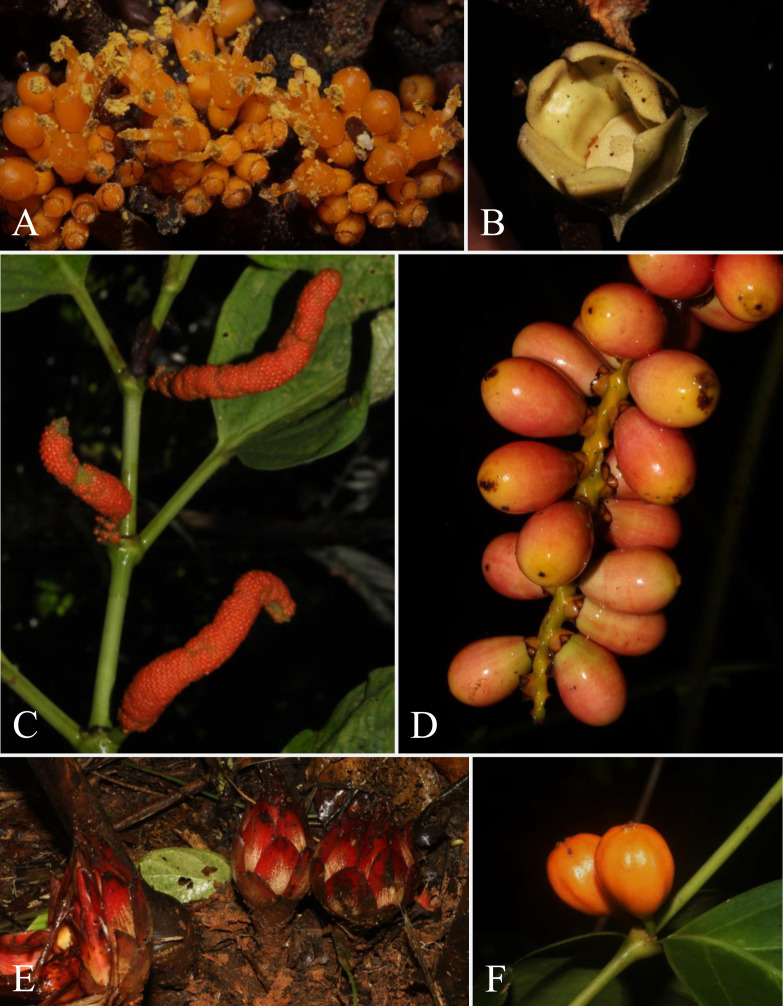
Selected Philippine endemic species occurring in SINP. **A** *Osmoxylon trilobatum*; **B** *Monoon mindanaense*; **C** *Piper brevicuspe*; **D** *Pinanga copelandii*; **E** *Hornstedtia conoidea*; **F** *Diplospora sorsogonensis*. Photographs by NKGA, SGSZ and RVAD.

The list includes 702 angiosperms, 167 ferns and allies and five gymnosperms. Rubiaceae remains the most species-rich family (95), followed by Orchidaceae (57), Moraceae (28), Polypodiaceae and Euphorbiaceae (27), Zingiberaceae and Arecaceae (24) (Fig. 4). Of the 845 taxa identified to the species level, 98.22% (830 species) are native. Furthermore, 339 are Philippine endemics (see Figs 5, 6, 7) and 37 are island-restricted species.

**Figure 8.**
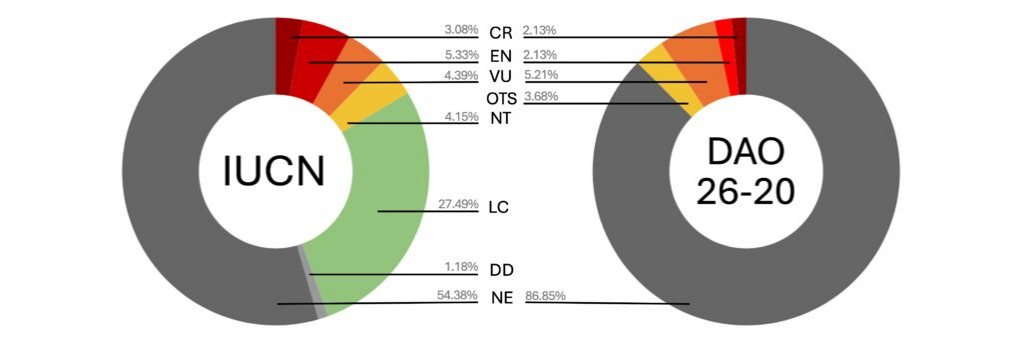
The conservation status, based on the IUCN Red List and DENR-DAO 2026-20, of identified vascular plant species in SINP in percentages (Abbreviations: CR - Critically Endangered, EN - Endangered, VU - Vulnerable, OTS - Other Threatened Species, NT - Near Threatened, LC - Least Concern, DD - Data Deficient, NE - Not Evaluated).

Based on the IUCN Red List and provisional assessments, 108 species (12.78%) are threatened (Vulnerable to Critically Endangered), 277 (32.78%) are Near Threatened, Least Concern or Data Deficient and 460 (54.44%) remain Not Evaluated. Under the national listing (DAO 2026-20), 80 (9.47%) are threatened (Vulnerable to Critically Endangered), 31 (3.67%) are Other Threatened Species, while 734 (86.86%) are Not Evaluated. Fig. 8 provides a summary of the conservation status by percentage.

## Discussion

As the largest terrestrial protected area in the Philippines, SINP harbours exceptional botanical diversity. Our consolidated checklist accounts for 887 vascular plant species, including 338 taxa found nowhere else in the country or the world. By integrating recent field collections with historical records and published accounts, this study provides a significant framework currently available for SINP and establishes an important baseline for future floristic monitoring, ecological plot-based studies and long-term biodiversity assessments for the Park. The predominance of large families, such as Rubiaceae and Orchidaceae, aligns with national trends reported by Pelser et al. (2011), which recognised both families as the most species-rich in the Philippine flora. Other dominant families in the country, such as Apocynaceae, Begoniaceae, Euphorbiaceae and Zingiberaceae, contribute substantially to the floristic richness of SINP. This pattern is further reflected in the localised survey of Paranas within SINP by Villanueva et al. (2021a) and is consistent with other regional floras across the archipelago (e.g. Amoroso et al. (2018), Batuyong et al. (2020), Romeroso et al. (2024)). At a broader biogeographical scale, the dominance of Orchidaceae and Rubiaceae also reflects SINP’s position within the Sunda–Sahul Convergence Zone, a region known for high diversity in these families (Joyce et al. 2020).

The documentation of numerous locality records for SINP and Samar as a whole provides range extensions for several species and contributes valuable biogeographical information for the Philippine flora. These new distributional data demonstrate that large portions of the SINP remain incompletely explored, especially in the most interior and remote areas of the Park. Moreover, several collections that were not identified up to the species level do not alone represent specimens lacking diagnosable features, but also represent potentially undescribed taxa, indicating that the true botanical diversity of SINP remains far from being fully characterised. These results confirm the identity of SINP and the Philippines more broadly, as a plant diversity darkspot (Ondo et al. 2024), where both Wallacean and Linnaean shortfalls persist. However, efforts to address these knowledge shortfalls are constrained by heightened security risks, as Samar is amongst the islands in the Visayas Region with elevated armed conflict and insurgent activity next to Mindanao (Holden 2013, Hilario-Husain et al. 2024), limiting access to the most remote and botanically promising areas of the Park.

SINP, characterised by its extensive karst landscapes, supports an exceptionally high concentration of Philippine endemic species, including several taxa known only from within the protected area, effectively functioning as an ark for the country's unique botanical heritage (Clements et al. 2006, Restificar et al. 2006) with their highly heterogeneous structures and strong environmental gradients promoting ecological specialisation and extremely narrow geographic ranges, traits that heighten the vulnerability of their associated species to local extinction. This risk is further amplified for Samar endemic species, given that SINP has experienced amongst the highest rates of forest loss in the country over recent decades (Apan et al. 2017), further constricting already limited habitat. Moreover, a substantial portion of the recorded species are classified as threatened under both the IUCN Red List and the national listing by DENR-DAO 2026-20, with the majority remaining as 'Not Evaluated'. The absence of formal conservation assessments for endemic species and the lack of data (Data Deficient) have tangible consequences. They often receive little to no conservation attention and may be excluded from policy-making and management priorities, especially for plants, as most conservation assessments and funding efforts are focused on higher vertebrates (Balding and Williams 2016, Edgar 2025). Collectively, these patterns exemplify the defining features of the Philippines as a priority hotspot, characterised by immense levels of endemism, alarming threats and knowledge shortfalls, with SINP standing out as a critical refuge for safeguarding many of the nation's most unique and vulnerable plant lineages.

A century after Merrill's celebrated work laid the foundation for documenting the nation's flora, this study reinforces both the progress achieved and the gaps that remain in understanding the botanical diversity of the Philippines. The updated inventory of SINP reveals that, despite decades of taxonomic advancements, large portions of the Philippine flora, particularly in its most remote areas, remain unexplored and undocumented. By integrating extensive fieldwork with diverse secondary sources, this study addresses critical shortfalls, revealing new distributional records, potential new species and exceptionally high levels of endemism. These findings not only update Merrill's century-old call for continued botanical exploration, but also urge sustained fieldwork in biologically complex and threatened landscapes such as SINP. Beyond its taxonomic and biogeographical contributions, this updated checklist establishes a reference framework for further ecological and conservation studies. More importantly, the floristic evidence presented here provides a robust scientific foundation for SINP's nomination as a UNESCO World Heritage Site, demonstrating its exceptional biodiversity, ecological uniqueness and its invaluable contribution to the Outstanding Universal Value of the global natural heritage.

## Supplementary Material

XML Treatment for Tracheophyta

XML Treatment for Lycopodiopsida, Polypodiopsida, Cycadopsida, Gnetopsida, Pinopsida, Liliopsida and Magnoliopsida

## Figures and Tables

**Table 1.** A checklist of vascular plants in SINP arranged by family. Summary: Taxon = scientific name of the taxon, species with * are new locality records; Endemicity = species' endemic status in the Philippines (abbreviations: N/E - Non-endemic, E - Endemic, SE - Samar Island endemic); IUCN and DAO 2026-20 = Conservation status for IUCN (global) and DAO (national) (abbreviations: NE - Not Evaluated, DD - Data Deficient, OTS - Other Threatened Species, LC - Least Concern, NT - Near Threatened, VU - Vulnerable, EN Endangered, CR - Critically Endangered, status with ^@^ are proposed conservation status from published literatures); Source Material = source of the species record (abbreviations: A - Villanueva et al. (2021a), B - Quimio (2016), C - Villanueva et al. (2022), D - Pelser et al. (2011), E - GBIF.org (2025a), GBIF.org (2025b), GBIF.org (2025c), F - Madera et al. (2021), G - delos Angeles et al. (2022), H - Adorador and Fernando (2017), I - Adorador and Fernando (2019), J - Obeña et al. (2021), K - Ordas et al. (2019), L - Chavez et al. (2020), M - Meneses et al. (2018), N - Meneses and Cootes (2019), O - Tandang et al. (2022), P - delos Angeles et al. (2022a), Q - delos Angeles et al. (2024), R - Fontarum-Bulawin et al. (2024), S - SINP Collection, T - Tandang et al. (2019), U - delos Angeles et al. (2022b), V - delos Angeles et al. (2023), W - Docot et al. (2026), X - Ordas et al. (2022), Y - Adorador (2019), Z - Adorador et al. (2021)).

**Taxon**	**Endemicity**	**IUCN Status**	**DAO 2026-20**	**Source Material**
** Acanthaceae **				
*Gymnostachyum affine* Nees	N/E	NE	NE	A
*Pseuderanthemum curtatum* (C.B.Clarke) Merr.	E	NE	NE	E
*Ptyssiglottis undulata* (Merr.) B.Hansen	E	NE	NE	E
*Rhaphidospora luzonensis* (C.B.Clarke) Bremek.	E	NE	NE	S, E
*Rungia membranacea* Merr.	SE	NE	NE	S
*Staurogyne samarensis* Bremek.	SE	NE	NE	E
*Strobilanthes bulusanensis* Elmer ex Y.F.Deng	E	NE	NE	D, A, E
*Strobilanthes reptans* (G.Forst.) Moylan ex Y.F.Deng & J.R.I.Wood	N/E	NE	NE	S
** Achariaceae **				
*Hydnocarpus subfalcata* Merr.	N/E	LC	NE	B
** Actinidiaceae **				
*Saurauia clementis* Merr.	E	LC	NE	S, E
*Saurauia latibractea* Choisy*	E	LC	NE	S
*Saurauia luzoniensis* Merr.*	E	NT	NE	S
*Saurauia samarensis* Merr.	SE	EN	NE	S
*Saurauia* sp.	-	-	-	S
** Amaranthaceae **				
Cyathula prostrata var. lancifolia (Merr.) Backer	E	NE	NE	E
** Anacardiaceae **				
*Buchanania nitida* Engl.	N/E	LC	NE	E
*Dracontomelon dao* (Blanco) Merr. & Rolfe	N/E	LC	OTS	S, B
*Koordersiodendron pinnatum* (Blanco) Merr.	N/E	VU	OTS	S
*Mangifera monandra* Merr.	E	NT	VU	A
*Semecarpus cuneiformis* Blanco	N/E	LC	NE	B, E
*Spondias pinnata* (L.f.) Kurz	N/E	LC	NE	E
** Annonaceae **				
*Cananga odorata* (Lam.) Hook.f. & Thomson	N/E	LC	NE	E
*Drepananthus acuminatus* (C.B.Rob.) Survesw. & R.M.K.Saunders	N/E	VU	NE	F
*Drepananthus apoensis* Elmer	E	VU	NE	B
*Drepananthus crassipetalus* (R.J.Wang & R.M.K.Saunders) Survesw. & R.M.K.Saunders	SE	EN	OTS	D, E
*Drepananthus samarensis* (R.J.Wang & R.M.K.Saunders) Survesw. & R.M.K.Saunders	SE	EN	OTS	E
*Goniothalamus amuyon* (Blanco) Merr.	N/E	VU	NE	E
*Goniothalamus elmeri* Merr.	E	LC	NE	S, E
*Goniothalamus* sp.	-	-	-	S
*Goniothalamus lancifolius* Merr.	E	EN	NE	D, A
*Meiogyne virgata* (Blume) Miq.	N/E	LC	NE	E
*Mitrephora pictiflora* Elmer	E	EN	NE	S
*Monoon mindanaense* (Elmer) B.Xue & R.M.K.Saunders*	E	EN	NE	S
*Monoon oblongifolium* (C.B.Rob.) B.Xue & R.M.K.Saunders	E	EN	NE	B
*Orophea cumingiana* S. Vidal	E	NT	NE	D, A
*Phaeanthus ophthalmicus* (Roxb. ex G.Don) J.Sinclair	N/E	LC	NE	E
*Polyalthia obliqua* Hook.f. & Thomson	N/E	LC	NE	S
*Pseuduvaria macgregorii* Merr.	SE	NE	NE	E
*Uvaria littoralis* (Blume) Blume	N/E	NE	NE	S
*Uvaria monticola* Miq.	N/E	NE	NE	S
** Apocynaceae **				
*Alstonia scholaris* (L.) R.Br.	N/E	LC	NE	B
*Cerbera manghas* L.	N/E	LC	NE	E
*Dischidia elmeri* Schltr.	E	DD^@^	OTS	S
*Dischidia hirsuta* (Blume) Decne.	N/E	NE	NE	S
*Dischidia major* (Vahl) Merr.*	N/E	NE	NE	S
*Dischidia nummularia* R.Br.*	N/E	NE	NE	S
*Dischidia purpurea* Merr.	E	NE	NE	S
*Hoya benguetensis* Schltr.	E	NE	NE	S
*Hoya cutis-porcelana* W.Suarez, J.R.Sahagun & Aurigue	E	NE	NE	S
*Hoya madulidii* Kloppenb.	E	NE	NE	S, E
*Hoya mitrata* Kerr*	E	NE	NE	S
*Hoya obscura* Elmer ex C.M.Burton*	N/E	NE	NE	S
*Hoya pentaphlebia* Merr.	E	NE	NE	S
*Kibatalia merrilliana* Woodson	E	EN	VU	A
*Kibatalia puberula* Merr.	SE	EN	EN	A, D
*Lepiniopsis ternatensis* Valeton	N/E	LC	NE	E
*Rauvolfia sumatrana* Jack	N/E	LC	NE	E
*Tabernaemontana macrocarpa* Jack	N/E	LC	NE	S
*Tabernaemontana pandacaqui* Poir.	N/E	LC	NE	S
*Vincetoxicum* sp.	-	-	-	S
Wrightia pubescens subsp. laniti (Blanco) Ngan	N/E	LC	NE	B
** Araceae **				
*Aglaonema commutatum* Schott	N/E	NE	NE	S
*Aglaonema densinervium* Engl.	N/E	NE	NE	E
*Aglaonema philippinense* Engl.	N/E	NE	NE	S
*Aglaonema simplex* Blume	N/E	LC	NE	E
*Alocasia heterophylla* (C.Presl) Merr.	E	NE	NE	S
*Alocasia sinuata* N.E.Br.	E	CR	NE	S
*Alocasia zebrina* G.W.Johnson & R.Hogg	E	NE	VU	S, A
*Amorphophallus longispathaceus* Engl. & Gehrm.	E	NE	NE	E
*Amorphophallus samarensis* Bulawin, Medecilo & Alejandro	SE	CR^@^	NE	R
*Cyrtosperma merkusii* (Hassk.) Schott	N/E	NE	NE	S
*Homalomena philippinensis* Engl.	N/E	NE	NE	S, A, J
*Pothos cylindricus* C.Presl	N/E	NE	NE	S
*Pothos dolichophyllus* Merr.	E	NE	NE	S
*Pothos ovatifolius* Engl.	N/E	NE	NE	S
*Rhaphidophora korthalsii* Schott	N/E	NE	NE	S, E
*Rhaphidophora minor* Hook.f.*	N/E	NE	NE	S
*Schismatoglottis calyptrata* (Roxb.) Zoll. & Moritzi	N/E	NE	NE	S
*Schismatoglottis edanoi* A.Hay	SE	NE	NE	E
*Schismatoglottis minuta* Tandang & M.D.Angeles	SE	CR^@^	CR	S, V
*Schismatoglottis samarensis* A.Hay	SE	NE	NE	S
*Spathiphyllum commutatum* Schott	N/E	NE	NE	S, E
** Araliaceae **				
*Heptapleurum* sp. 1	-	-	-	S
*Heptapleurum* sp. 2	-	-	-	S
*Heptapleurum* sp. 3	-	-	-	S
*Heptapleurum insularum* Seem.	E	LC	NE	E
*Osmoxylon oblongifolium* Philipson	E	NE	NE	S
*Osmoxylon trilobatum* (Merr.) Philipson	E	NT	NE	S
*Polyscias aherniana* (Merr.) Lowry & G.M.Plunkett	N/E	LC	NE	S
*Polyscias nodosa* (Blume) Seem.	N/E	LC	NE	A, C
** Araucariaceae **				
*Agathis dammara* (Lamb.) Rich.	N/E	VU	VU	S, B
** Arecaceae **				
*Areca ipot* Becc.*	E	EN	VU	S
*Calamus aidae* Fernando	E	NE	VU	S, H, D, A
*Calamus discolor* C. Mart.	E	NE	NE	D, A
Calamus erinaceus subsp. daemonoropoides (Fernando) A.J.Hend.	E	LC	VU	H
*Calamus filispadix* Becc.	E	NE	NE	H
*Calamus loherianus* (Becc.) W.J.Baker	E	NE	CR	H
Calamus oblongus subsp. mollis (Blanco) A.J.Hend.	N/E	NE	CR	H
*Calamus ochrolepis* (Becc.) W.J. Baker	E	NE	NE	D, A
*Calamus symphysipus* C. Mart.	N/E	NE	NE	D, A
*Calamus warayanus* Adorndor & Fernando	SE	CR^@^	CR	H, A
Calamus zollingeri subsp. merrillii (Becc.) A.J.Hend.	E	NE	NE	H, A
*Caryota cumingii* Lodd. ex Mart.	E	LC	NE	C
*Caryota rumphiana* Mart.	N/E	LC	NE	A, C
*Heterospathe elmeri* Becc.	E	EN	NE	E
*Heterospathe fernandoi* Adorador	E	EN	EN	Y
*Heterospathe intermedia* (Becc.) Fernando	E	VU	NE	H, J, A, C
*Korthalsia laciniosa* (Griff.) Mart.	N/E	NE	NE	S
*Oncosperma tigillarium* (Jack) Ridl.	N/E	LC	VU	C
*Orania zheae* Adorador & Fernando	SE	CR^@^	NE	I, A
*Pinanga copelandii* Becc.	E	NE	NE	S, D, A
*Pinanga gruezoi* Adorador & Fernando	E	EN^@^	EN	A
*Pinanga samarana* Becc.	E	EN	CR	H
Plectocomia elongata var. philippinensis Madulid	E	NE	OTS	H
*Saribus rotundifolius* (Lam.) Blume	N/E	LC	OTS	J
** Aristolochaceae **				
*Thottea tomentosa* (Blume) Ding Hou	N/E	CR^@^	NE	D, A
** Asparagaceae **				
*Dracaena angustifolia* (Medik.) Roxb.	N/E	NE	NE	S, C
** Asphodelaceae **				
*Dianella ensifolia* (L.) Redouté	N/E	NE	NE	S
** Aspleniaceae **				
*Asplenium affine* Sw.	N/E	NE	NE	S, G
*Asplenium anguineum* Christ	N/E	NE	NE	G
*Asplenium apoense* Copel.	E	NE	NE	S, G
*Asplenium laserpitiifolium* Lam.	N/E	NE	NE	G, E
*Asplenium nidus* L.	N/E	NE	NE	S, G
*Asplenium persicifolium* J.Sm. ex Mett.	N/E	NE	NE	S, G
*Asplenium phyllitidis* D.Don	N/E	NE	NE	G, E
*Asplenium scolopendrioides* J.Sm. ex Hook.	N/E	NE	NE	G
*Asplenium tenerum* G.Forst.	N/E	NE	NE	S, G, E
*Asplenium vulcanicum* Blume	N/E	NE	NE	G
** Asteraceae **				
*Blumea balsamifera* (L.) DC.	N/E	LC	NE	S
*Crassocephalum crepidioides* (Benth.) S.Moore	N/E	NE	NE	E
*Elephantopus mollis* Kunth	N/E	NE	NE	E
** Athyriaceae **				
*Diplazium cordifolium* Blume	N/E	NE	NE	S
*Diplazium cumingii* (C.Presl) C.Chr.	N/E	NE	NE	S
*Diplazium symmetricum* (Copel.) M.G.Price	SE	NE	NE	G
*Diplazium vestitum* C.Presl	N/E	NE	OTS	G
** Balsaminaceae **				
*Impatiens platypetala* Lindl.	N/E	NE	NE	S, E
** Begoniaceae **				
*Begonia biliranensis* Merr.	E	EN^@^	NE	S
*Begonia burabod* Rubite, C.Justo, P.Villaseñor & C.W.Lin	SE	LC^@^	NE	S
*Begonia colorata* Warb.	E	VU^@^	NE	S
*Begonia jagorii* Warb.*	E	CR^@^	NE	S
*Begonia* sp. 1	-	-	-	S
*Begonia* sp. 2	-	-	-	S
*Begonia* sp. 3	-	-	-	S
*Begonia longistipula* Merr.	E	EN^@^	NE	S, E
*Begonia normaaguilariae* M.D.Angeles, Rubite & Tandang	SE	CR^@^	NE	S, U
*Begonia quercifolia* A.DC.	E	DD^@^	NE	S
*Begonia samarensis* Merr.	SE	DD^@^	NE	S, E
*Begonia sohoton* Rubite, C.Justo, P.Villaseñor & C.W.Lin	SE	VU^@^	VU	S
** Blechnaceae **				
Austroblechnum melanocaulon subsp. melanocaulon (Brack.) Gasper & V.A.O.Dittrich	N/E	NE	NE	E
*Blechnopsis orientalis* (L.) C.Presl	N/E	NE	NE	G
*Oceaniopteris egregia* (Copel.) Gasper & Salino	N/E	NE	VU	S, A
*Stenochlaena milnei* Underw.	N/E	NE	NE	S
*Stenochlaena palustris* (Burm.f.) Bedd.	N/E	NE	NE	S
** Burseraceae **				
Canarium euryphyllum var. ramosii (Merr.) Leenh.*	E	LC	NE	S
*Canarium hirsutum* Willd.	N/E	LC	NE	E, B, A, C
*Canarium luzonicum* (Blume) A.Gray	N/E	NT	OTS	B
*Canarium ovatum* Engl.	E	LC	NE	E, B
** Buxaceae **				
*Buxus rolfei* S.Vidal	E	LC	NE	E
** Calophyllaceae **				
*Calophyllum blancoi* Planch. & Triana	N/E	LC	NE	S, E, B
*Calophyllum inophyllum* L.	N/E	LC	NE	E
*Calophyllum soulattri* Burm.f.	N/E	LC	NE	C
** Campanulaceae **				
*Hippobroma longiflora* (L.) G.Don	N/E	NE	NE	E
** Cannabaceae **				
*Celtis philippensis* Blanco	N/E	LC	NE	S
*Gironniera celtidifolia* Gaudich.	N/E	LC	NE	S, E
*Gironniera subaequalis* Planch.	N/E	LC	NE	E
** Cardiopteridaceae **				
*Gonocaryum calleryanum* (Baill.) Becc.	N/E	LC	NE	S
** Casuarinaceae **				
*Gymnostoma rumphianum* (Miq.) L.A.S.Johnson	N/E	LC	NE	A, C
** Centroplacaceae **				
*Bhesa paniculata* Arn.	N/E	LC	NE	E
** Chloranthaceae **				
*Chloranthus officinalis* Blume	N/E	NE	NE	E
** Clusiaceae **				
*Garcinia nervosa* (Miq.) Miq.	N/E	LC	NE	S
*Garcinia oligophlebia* Merr.	E	NE	NE	B
*Garcinia rubra* Merr.	E	NT	NE	E, A, C
*Garcinia venulosa* (Blanco) Choisy	E	LC	NE	B
*Garcinia vidalii* Merr.	E	LC	NE	S
** Combretaceae **				
*Combretum indicum* (L.) DeFilipps	N/E	NE	NE	E
*Terminalia macrantha* Rojo	SE	CR	VU	F
*Terminalia microcarpa* Decne.	N/E	LC	NE	S
** Commelinaceae **				
*Amischotolype hispida* (A.Rich.) D.Y.Hong	N/E	NE	NE	S
*Pollia secundiflora* (Blume) Bakh.f.	N/E	NE	NE	S
*Pollia thyrsiflora* (Blume) Steud.	N/E	NE	NE	S
** Connaraceae **				
*Agelaea borneensis* (Hook.f.) Merr.	N/E	LC	NE	E
*Agelaea trinervis* (Llanos) Merr.	N/E	LC	NE	E
Connarus monocarpus subsp. malayensis Leenh.	N/E	NE	NE	E
** Convolvulaceae **				
*Decalobanthus peltatus* (L.) A.R.Simões & Staples	N/E	NE	NE	E
** Cornaceae **				
*Alangium longiflorum* Merr.	N/E	LC	OTS	E
*Alangium pilosum* Merr.	N/E	EN	NE	E
** Costaceae **				
*Hellenia speciosa* (J.Koenig) S.R.Dutta	N/E	LC	NE	S
** Ctenolophonaceae **				
*Ctenolophon parvifolius* Oliv.	N/E	VU	VU	B
** Cyatheaceae **				
*Alsophila fuliginosa* Christ	E	NE	VU	G
*Sphaeropteris integra* (J.Sm. ex Hook.) R.M.Tryon	N/E	NE	NE	S
*Sphaeropteris polypoda* (Baker) R.M.Tryon	N/E	NE	NE	G
** Cycadaceae **				
*Cycas riuminiana* M.Porte ex Regel	E	EN	VU	C
** Cyperaceae **				
*Cyperus cyperoides* (L.) Kuntze	N/E	LC	NE	E
*Cyperus iria* L.	N/E	LC	NE	E
*Fimbristylis dichotoma* (L.) Vahl	N/E	LC	NE	E
*Hypolytrum compactum* Nees & Meyen ex Kunth*	N/E	NE	NE	S
*Hypolytrum nemorum* (Vahl) Spreng.	N/E	LC	NE	E
** Davalliaceae **				
*Davallia embolostegia* Copel.	N/E	NE	NE	G
*Davallia falcinella* C.Presl	E	NE	NE	G
*Davallia heterophylla* Sm.	N/E	NE	NE	G
*Davallia pectinata* Sm.	N/E	NE	NE	G
*Davallia repens* (L.f.) Kuhn	N/E	NE	NE	G, E
*Davallia solida* (Forst.) Sw.	N/E	NE	NE	G
*Davallia trichomanoides* Blume*	N/E	NE	NE	S
*Davallodes hirsuta* (C.Presl) Copel.	N/E	NE	NE	G
** Dilleniaceae **				
*Dillenia indica* L.	N/E	LC	NE	S
*Dillenia philippinensis* Rolfe	E	NT	NE	S, E, B
** Dioscoreaceae **				
*Dioscorea hispida* Dennst.	N/E	NE	NE	E
*Tacca palmata* Blume	N/E	NE	NE	S
** Dipteridaceae **				
*Dipteris conjugata* Reinw.	N/E	NE	NE	G
** Dipterocarpaceae **				
*Anisoptera thurifera* (Blanco) Blume	N/E	VU	NE	B
*Anthoshorea assamica* (Dyer) P.S.Ashton & J.Heck.	N/E	NE	NE	B
*Dipterocarpus gracilis* Blume	N/E	VU	VU	B
*Dipterocarpus grandiflorus* (Blanco) Blanco	N/E	EN	VU	B
*Dipterocarpus validus* Blume	N/E	LC	NE	B
*Hopea foxworthyi* Elmer	E	EN	CR	B
*Hopea malibato* Foxw.	E	VU	CR	B
*Hopea philippinensis* Dyer	E	EN	CR	S, F, A, C
*Hopea quisumbingiana* H.G.Gut.	E	EN	CR	S, A
*Hopea samarensis* H.G.Gut.	E	EN	CR	A
*Parashorea malaanonan* (Blanco) Merr.	N/E	LC	NE	E, B
*Pentacme contorta* (S.Vidal) Merr. & Rolfe	E	LC	VU	S, C
*Rubroshorea almon* (Foxw.) P.S.Ashton & J.Heck.	N/E	NT	VU	F, B
*Rubroshorea negrosensis* (Foxw.) P.S.Ashton & J.Heck.	E	LC	VU	S, F, B, A, C
*Rubroshorea palosapis* (Blanco) P.S.Ashton & J.Heck.	E	LC	NE	S, F, B
*Rubroshorea polysperma* (Blanco) P.S.Ashton & J.Heck.	E	LC	VU	S, F, B
*Shorea astylosa* Foxw.	E	EN	EN	F, B, C
Shorea falciferoides subsp. falciferoides Foxw.	E	VU	VU	B
*Shorea guiso* (Blanco) Blume	N/E	VU	NE	F
*Vatica mangachapoi* Blanco	N/E	VU	NE	C
** Dryopteridaceae **				
*Arthrobotrya articulata* (J.Sm. ex Fée) J.Sm.	N/E	NE	NE	E
*Bolbitis heteroclita* (C.Presl) Ching	N/E	NE	NE	S, G, E
*Bolbitis quoyana* (Gaud.) Ching	N/E	NE	NE	G, E
*Bolbitis sinuata* (C.Presl) Hennipman	N/E	NE	NE	S, G, E
*Ctenitis pallens* (Brack.) M.G.Price	N/E	NE	NE	G, E
*Ctenitis setosa* (C.Presl) Holttum	E	NE	NE	G
*Ctenitis silvatica* Holttum	N/E	NE	NE	G
*Dryopteris subarborea* (Baker) C.Chr.	N/E	NE	NE	G, E
*Elaphoglossum luzonicum* Copel.	N/E	NE	NE	G, E
*Elaphoglossum melanostictum* (Blume) T.Moore	N/E	NE	NE	G, E
*Lomagramma copelandii* Holttum	N/E	NE	NE	G, E
*Pleocnemia cumingiana* C.Presl	N/E	NE	NE	E
*Pleocnemia macrodonta* (Fée) Holttum	N/E	NE	NE	G, E
*Polystichum moluccense* T. Moore	N/E	NE	VU	A
*Teratophyllum articulatum* (Hook.) Mett. ex Kuhn	N/E	NE	NE	D, G
*Teratophyllum koordersii* Holttum	N/E	NE	NE	S, G, E
*Teratophyllum leptocarpum* (Fée) Holttum	N/E	NE	NE	S, G
** Ebenaceae **				
*Diospyros blancoi* A.DC.	N/E	NE	NE	S, F, C
*Diospyros montana* Roxb.	N/E	LC	NE	B
*Diospyros philippinensis* A.DC.	N/E	NT	OTS	B
*Diospyros pyrrhocarpa* Miq.	N/E	LC	VU	F
** Ehretiaceae **				
*Ehretia microphylla* Lam.*	N/E	NE	NE	S
** Elaeocarpaceae **				
*Elaeocarpus leytensis* Merr.	E	CR	NE	B
*Elaeocarpus multiflorus* Fern.-Vill.	N/E	NE	NE	E
*Elaeocarpus octopetalus* Merr.	N/E	LC	NE	B
** Ericaceae **				
*Vaccinium perrigidum* Elmer	E	NE	NE	B
** Erythropalaceae **				
*Erythropalum scandens* Blume	N/E	LC	NE	S
** Euphorbiaceae **				
*Acalypha amentacea* Roxb.	N/E	NE	NE	S, E
*Agrostistachys borneensis* Becc.	N/E	LC	NE	E
*Alchornea rugosa* (Lour.) Müll.Arg.	N/E	LC	NE	S, E
*Balakata luzonica* (S.Vidal) Esser	N/E	LC	OTS	F, B
*Blumeodendron kurzii* (Hook.f.) J.J.Sm.	N/E	LC	NE	E
*Blumeodendron philippinense* Merr. & Rolfe	E	NE	NE	B
*Claoxylon albicans* (Blanco) Merr.	E	NT	NE	E
*Claoxylon pubescens* Quisumb.	E	EN	NE	B
*Claoxylon subviride* Elmer	E	NE	NE	E
*Cleidion ramosii* (Merr.) Merr.	E	NE	NE	E
*Codiaeum luzonicum* Merr.*	E	NE	NE	S
*Codiaeum macgregorii* Merr.	E	CR	NE	A, C
*Croton consanguineus* Müll.Arg.	E	NE	NE	B, E
*Excoecaria philippinensis* Merr.	N/E	LC	NE	S
*Hancea wenzeliana* (Slik) S.E.C.Sierra, Kulju & Welzen	E	CR	NE	A, C
*Homonoia riparia* Lour.	N/E	LC	NE	E
*Macaranga bicolor* Müll.Arg.	E	LC	NE	A, C
*Macaranga hispida* (Blume) Müll.Arg.	N/E	LC	NE	S
*Macaranga leytensis* Merr.*	E	EN	NE	S
*Macaranga ovatifolia* Merr.	N/E	EN	NE	S
*Mallotus cumingii* Müll.Arg.	N/E	LC	NE	B
*Manihot esculenta* Crantz	N/E	NE	NE	E
*Reutealis trisperma* (Blanco) Airy Shaw	E	NT	VU	B
*Spathiostemon javensis* Blume	N/E	LC	NE	S, E
*Suregada glomerulata* (Blume) Baill.	N/E	LC	NE	S
Trigonostemon villosus var. merrillianus (Airy Shaw) R.Y.Yu & Welzen	N/E	NE	NE	E
*Tritaxis ixoroides* (C.B.Rob.) R.Y.Yu & Welzen	E	VU	NE	A, C
** Fabaceae **				
*Afzelia rhomboidea* (Blanco) Fern.-Vill.	N/E	LC	EN	B
*Albizia saponaria* (Lour.) Blume ex Miq.	N/E	LC	NE	B
*Archidendron clypearia* (Jack) I.C.Nielsen	N/E	LC	NE	E
*Archidendron fagifolium* (Blume ex Miq.) I.C.Nielsen	N/E	LC	NE	E
Archidendron pauciflorum var. caulostachyum (Merr.) I.C.Nielsen	SE	NE	NE	E
*Calliandra haematocephala* Hassk.	N/E	LC	NE	B
*Cynometra inaequifolia* A.Gray	E	NT	OTS	B
*Ormosia calavensis* Azaola	N/E	LC	NE	B
*Phanera aherniana* (G.Perkins) de Wit*	N/E	NE	VU	S
*Pleurolobus gangeticus* (L.) J.St.-Hil. ex H.Ohashi & K.Ohashi*	N/E	NE	NE	S
*Prioria alternifolia* (Elmer) Breteler	N/E	LC	OTS	F
*Pterocarpus indicus* Willd.	N/E	EN	VU	B
*Wallaceodendron celebicum* Koord.	N/E	NT	OTS	F, B, A, C
** Fagaceae **				
*Lithocarpus celebicus* (Miq.) Rehder	N/E	LC	NE	B
*Lithocarpus solerianus* (Vidal) Rehder	E	LC	NE	E
** Flagellariaceae **				
*Flagellaria indica* L.	N/E	NE	NE	S, E
** Gentianaceae **				
*Fagraea auriculata* Jack	N/E	LC	NE	E
*Fagraea ceilanica* Thunb.	N/E	LC	NE	E
*Utania volubilis* (Wall.) Sugumaran*	N/E	LC	NE	S
** Gesneriaceae **				
*Aeschynanthus cardinalis* (H.F.Copel. ex Merr.) Schltr.*	E	NE	NE	S
*Cyrtandra* sp.	-	-	-	S
*Cyrtandra pallidifolia* Kraenzl.	E	NE	NE	S
*Cyrtandra samarensis* Tandang & M.D.Angeles	SE	LC^@^	NE	Q
*Monophyllaea merrilliana* Kraenzl.	N/E	NE	OTS	S
*Rhynchoglossum klugioides* C.B.Clarke	N/E	NE	NE	E
*Rhynchotechum discolor* (Maxim.) B.L.Burtt	N/E	LC^@^	NE	E
** Gleicheniaceae **				
*Dicranopteris linearis* (Burm.f.) Underw.	N/E	LC	NE	G
*Dicranopteris taiwanensis* Ching & Chiu	N/E	NE	NE	S
** Gnetaceae **				
*Gnetum gnemon* L.	N/E	LC	NE	S, E, B, C
*Gnetum latifolium* Blume	N/E	LC	NE	E
** Hydrocharitaceae **				
*Hydrilla verticillata* (L.f.) Royle*	N/E	LC	NE	S
** Hymenophyllaceae **				
*Abrodictyum cumingii* C.Presl	N/E	NE	NE	S, G
*Callistopteris apiifolia* (Presl) Copel.	N/E	NE	NE	G
*Cephalomanes atrovirens* C.Presl	N/E	NE	NE	G
*Cephalomanes crassum* (Copel.) M.G.Price	N/E	NE	NE	G, E
*Crepidomanes bipunctatum* (Poir.) Copel.	N/E	NE	NE	S
*Crepidomanes brevipes* (C.Presl) Copel.	N/E	NE	NE	S, G
*Crepidomanes christii* (Copel.) Copel.	N/E	NE	NE	G, E
*Crepidomanes humile* Bosch	N/E	NE	NE	G
*Didymoglossum mindorense* (Christ) K.Iwats.	N/E	NE	NE	G
*Hymenophyllum holochilum* (Bosch) C.Chr.	N/E	NE	NE	S, G
** Hypericaceae **				
*Cratoxylum formosum* (Jack) Benth. & Hook.f. ex Dyer	N/E	LC	NE	E
** Hypodematiaceae **				
*Leucostegia immersa* C.Presl	N/E	NE	NE	G, E
** Hypoxidaceae **				
*Curculigo capitulata* (Lour.) Kuntze	N/E	NE	NE	S
*Curculigo latifolia* Dryand. ex W.T.Aiton	N/E	NE	NE	S
** Icacinaceae **				
*Phytocrene macrophylla* (Blume) Blume	N/E	NE	NE	E
** Lamiaceae **				
*Clerodendrum brachyanthum* Schauer	E	LC	NE	E
Clerodendrum japonicum var. bethuneanum (H.Low) Wearn & Mabb.*	N/E	LC	NE	S
*Clerodendrum minahassae* Teijsm. & Binn.	N/E	LC	NE	E
*Hyptis brevipes* Poit.	N/E	NE	NE	E
*Hyptis capitata* Jacq.*	N/E	NE	NE	S
*Leucas decemdentata* (Willd.) Sm.*	N/E	NE	NE	S
*Premna oblongata* Miq.	N/E	LC	NE	S
*Premna serratifolia* L.	N/E	LC	NE	E
*Teijsmanniodendron ahernianum* (Merr.) Bakh.	N/E	LC	NE	E, F, B
*Teijsmanniodendron pteropodum* (Miq.) Bakh.	N/E	LC	NE	S, B, E
*Vitex parviflora* Juss.	N/E	LC	VU	S
*Vitex quinata* (Lour.) F.N.Williams	N/E	LC	NE	B
*Vitex turczaninowii* Merr.	N/E	LC	NE	A, C
** Lauraceae **				
*Actinodaphne dolichophylla* (Merr.) Merr.	E	VU	NE	B
*Actinodaphne tayabensis* (Elmer) Merr.	E	NT	NE	E
*Cinnamomum burmanni* (Nees & T.Nees) Blume	N/E	LC	NE	E
*Cinnamomum mercadoi* S.Vidal	E	LC	OTS	B
*Cryptocarya lauriflora* (Blanco) Merr.	E	NT	NE	E
*Cryptocarya oligocarpa* Merr.	E	VU	NE	B
*Litsea albayana* S.Vidal	E	NE	NE	B
*Litsea cordata* (Jack) Hook.fil.	N/E	LC	NE	E, B
*Litsea fulva* (Blume) Fern.-Vill.	N/E	LC	NE	S, E
*Litsea quercoides* Elmer	E	LC	NE	E
*Neolitsea villosa* (Blume) Merr.	N/E	LC	NE	E
*Nothaphoebe leytensis* (Elmer) Merr.	E	NE	NE	F, A
** Lecythidaceae **				
*Barringtonia macrostachya* (Jack) Kurz	N/E	LC	NE	S
*Petersianthus quadrialatus* Merr.	E	NT	NE	B
*Planchonia spectabilis* Merr.*	E	NT	NE	S
** Lindsaeaceae **				
*Lindsaea adiantoides* J.Sm. ex Hook.	E	NE	NE	G
*Lindsaea heterophylla* Dryand.	N/E	NE	NE	G
*Lindsaea pulchella* (J.Sm.) Mett. ex Kuhn	N/E	NE	NE	G
*Lindsaea rigida* J.Sm. ex Hook.	N/E	NE	NE	S
*Tapeinidium pinnatum* (Cav.) C.Chr.	N/E	NE	NE	G, E
** Loganiaceae **				
*Strychnos axillaris* Colebr.	N/E	NE	NE	E
*Strychnos minor* Dennst.	N/E	NE	NE	E
** Lomariopsidaceae **				
*Cyclopeltis crenata* (Fée) C.Chr.	N/E	NE	NE	S, A, G
*Cyclopeltis presliana* (J.Sm.) Berk.	N/E	NE	NE	G, E
*Lomariopsis lineata* (C.Presl) Holttum	N/E	NE	NE	G, E
*Lomariopsis subtrifoliata* Holttum	E	NE	NE	G, E
** Loranthaceae **				
*Amyema seriata* (Merr.) Barlow*	E	NE	NE	S
*Decaisnina confertiflora* (Merr.) Barlow	E	NE	NE	E
*Decaisnina cumingii* (Tiegh.) Barlow	N/E	NE	NE	S
*Decaisnina tomentosa* M.D.Angeles, Tandang, Caraballo & Buot	SE	DD^@^	OTS	O
** Lycopodiaceae **				
*Palhinhaea cernua* (L.) Vasc. & Franco	N/E	NE	NE	G
*Phlegmariurus salvinioides* (Hert.) Ching	N/E	NE	EN	G
** Lygodiaceae **				
*Lygodium auriculatum* (Willd.) Alston	N/E	NE	NE	S, G
*Lygodium japonicum* (Thunb.) Sw.	N/E	NE	NE	S
*Lygodium merrillii* Copel.	N/E	NE	NE	G
*Lygodium microphyllum* (Cav.) R.Br.	N/E	LC	NE	G
*Lygodium versteegii* Christ	N/E	NE	NE	G, E
** Malpighiaceae **				
*Aspidopterys elliptica* (Blume) A.Juss.	N/E	NE	NE	E
** Malvaceae **				
*Abelmoschus moschatus* Medik.	N/E	LC	NE	E
*Camptostemon philippinensis* (S.Vidal) Becc.	N/E	EN	EN	B
*Commersonia bartramia* (L.) Merr.	N/E	LC	NE	S
*Heritiera sylvatica* S.Vidal	N/E	LC	NE	B
*Hibiscus tiliaceus* L.	N/E	LC	NE	S
*Kleinhovia hospita* L.	N/E	LC	NE	E, C
*Microcos philippinensis* (Perkins) Burret	E	VU	NE	E
*Pterospermum obliquum* Blanco	E	LC	NE	S, B
*Sida acuta* Burm.f.*	N/E	NE	NE	S
*Trichospermum discolor* Elmer	E	VU	NE	B
*Trichospermum lanigerum* (Blanco) Merr.	E	NE	NE	S
** Marantaceae **				
*Donax canniformis* (G.Forst.) K.Schum.	N/E	NE	NE	E, S
*Phrynium bracteosum* (Warb. ex K.Schum.) Suksathan & Borchs.	N/E	NE	NE	S
*Phrynium fasciculatum* (C.Presl) Horan.	N/E	NE	NE	S
*Phrynium interruptum* (K.Schum.) Suksathan & Borchs.	N/E	NE	NE	S
*Phrynium minutiflorum* Suksathan & Borchs.	E	NE	VU	S, D, A
*Phrynium pubinerve* Blume	N/E	NE	NE	S
** Marattiaceae **				
*Angiopteris evecta* (G.Forst.) Hoffm.	N/E	NE	OTS	S, A, G
*Christensenia lobbiana* (de Vriese) Rolleri	N/E	NE	NE	G
*Ptisana pellucida* (C.Presl) Murdock	N/E	NE	NE	G, S, E
** Matoniaceae **				
*Phanerosorus major* Diels	N/E	NE	EN	S
** Melastomataceae **				
*Astronia megalantha* Merr.	E	NT	NE	S
*Medinilla cephalophora* Merr.	E	NE	NE	S, E
*Medinilla copelandii* Merr.*	E	NE	NE	S
*Medinilla magnifica* Lindl.*	E	NE	CR	S
*Medinilla malabrigoi* Z.D.Meneses, Adorador & Quakenbush	SE	EN^@^	VU	Z
*Medinilla multiflora* Mansf.	E	NE	NE	S, E
*Medinilla peltata* Merr.	E	NE	NE	S
*Medinilla polillensis* C.B.Rob	E	NE	NE	S
*Medinilla quadrifolia* (Blume) Blume	N/E	NE	NE	E
*Medinilla setigera* (Blume) Miq.	N/E	NE	NE	S, E
*Medinilla ternifolia* Triana*	E	NE	NE	S
*Medinilla teysmannii* Miq.	N/E	NE	NE	S, E
*Melastoma malabathricum* L.	N/E	NE	NE	S
*Memecylon sessilifolium* Merr.	E	NE	NE	B
** Meliaceae **				
*Aglaia argentea* Blume*	N/E	LC	NE	S
*Aglaia crassinervia* Kurz ex Hiern	N/E	LC	NE	E
*Aglaia leucophylla* King	N/E	LC	NE	B
*Aglaia pachyphylla* Miq.	N/E	LC	NE	E
*Aglaia rimosa* (Blanco) Merr.	N/E	LC	NE	A, C
*Aphanamixis polystachya* (Wall.) R.Parker	N/E	LC	NE	E
*Chisocheton cumingianus* (C.DC.) Harms	N/E	LC	NE	E, F, B
*Didymocheton mollissimus* (Spreng.) Mabb.	N/E	NE	NE	F
*Epicharis cuneata* (Hiern) Harms	N/E	LC	VU	E
*Goniocheton arborescens* Blume	N/E	LC	NE	E
*Reinwardtiodendron celebicum* Koord.	N/E	NE	NE	B
*Sandoricum koetjape* (Burm.f.) Merr.	N/E	LC	NE	B
*Toona calantas* Merr. & Rolfe	N/E	DD	VU	B
*Vavaea amicorum* Benth.	N/E	LC	NE	E, C
** Moraceae **				
*Artocarpus blancoi* (Elmer) Merr.*	E	LC	NE	S
*Artocarpus lamellosus* Blanco	E	NT	NE	B
*Artocarpus rubrovenius* Warb.	E	NE	NE	A, C
*Artocarpus treculianus* Elmer	E	LC	NE	E
*Ficus ampelas* Burm.fil.	N/E	LC	NE	S, E, A, C
*Ficus banahaensis* Elmer	E	LC	NE	C
*Ficus benjamina* L.	N/E	LC	NE	S
*Ficus botryocarpa* Miq.	N/E	LC	NE	S, E
*Ficus carpenteriana* Elmer	E	LC	NE	E
*Ficus congesta* Roxb.	N/E	LC	NE	S
*Ficus cumingii* Miq.	N/E	LC	NE	E
*Ficus fiskei* Elmer	E	LC	NE	S
*Ficus gigantifolia* Merr.	E	NT	NE	S
*Ficus gul* K.Schum. & Lauterb.	N/E	LC	NE	E
*Ficus heteropleura* Blume	N/E	NE	NE	E
*Ficus* sp. 1	-	-	-	S
*Ficus* sp. 2	-	-	-	S
*Ficus* sp. 3	-	-	-	S
*Ficus lepicarpa* Blume*	N/E	LC	NE	S
*Ficus minahassae* (Teijism. & Vriese) Miq.	N/E	LC	NE	S
*Ficus odorata* (Blanco) Merr.	E	LC	NE	S
*Ficus ruficaulis* Merr.	N/E	LC	NE	E
*Ficus sagittata* Vahl	N/E	LC	NE	E
*Ficus scaberrima* Blume	N/E	LC	NE	E
*Ficus septica* Burm.f.	N/E	LC	NE	E
*Ficus subulata* Blume	N/E	LC	NE	E
*Ficus sumatrana* (Miq.) Miq.	N/E	LC	NE	E
*Ficus ulmifolia* Lam.	E	LC	NE	S
** Musaceae **				
*Musa acuminata* Colla	N/E	LC	NE	E
** Myricaceae **				
*Myrica javanica* Blume	N/E	LC	NE	B
** Myristicaceae **				
*Endocomia macrocoma* (Miq.) W.J.de Wilde	N/E	LC	NE	E
*Horsfieldia ardisiifolia* (A.DC.) Warb.	E	VU	NE	A
*Horsfieldia samarensis* W.J.de Wilde	SE	CR	VU	A
*Knema glomerata* (Blanco) Merr.	N/E	LC	NE	E
Knema stellata subsp. stellata Merr.	SE	NT	NE	A
*Knema tomentella* (Miq.) Warb.	N/E	LC	NE	E
*Myristica agusanensis* Elmer	E	NT	NE	B
*Myristica cumingii* Warb.	E	LC	NE	E
*Myristica fragrans* Houtt.	N/E	DD	NE	B
*Myristica mindanaensis* Warb.	N/E	NE	NE	B
*Myristica philippensis* Lam.	E	LC	NE	S
*Myristica pilosigemma* W.J.dc Wilde	E	CR	OTS	A
** Myrtaceae **				
*Eugenia aherniana* C.B. Rob.	N/E	LC	NE	S
*Eugenia tulanan* Merr.	E	NE	NE	B, E
*Syzygium albayense* Merr.	E	CR	NE	B
*Syzygium crassibracteatum* (Merr.) Merr.	E	EN	NE	B
*Syzygium hutchinsonii* (C.B.Robinson) Merr.	E	CR	NE	B
*Syzygium polycephaloides* (C.B.Rob.) Merr.	N/E	NE	NE	S, B
*Syzygium simile* (Merr.) Merr.	N/E	NT	NE	F
*Syzygium striatulum* (C.B.Rob.) Merr.	E	VU	NE	B
*Tristaniopsis littoralis* (Merr.) Peter G.Wilson & J.T.Waterh.	E	EN	VU	S
*Tristaniopsis micrantha* (Merr.) Peter G.Wilson & J.T.Waterh.	E	EN	NE	F, B
*Xanthostemon philippinensis* Merr.	E	VU	CR	F
** Nepenthaceae **				
*Nepenthes alata* Blanco	E	LC	NE	E
*Nepenthes graciliflora* Elmer	E	LC	NE	E
*Nepenthes samar* Jebb & Cheek	SE	NE	CR	D, A
** Nephrolepidaceae **				
*Nephrolepis falcata* (Cav.) C.Chr.	N/E	NE	NE	G
** Ochnaceae **				
*Neckia serrata* Korth.	N/E	NE	OTS	E
** Olacaceae **				
*Anacolosa frutescens* (Blume) Blume	N/E	LC	NE	E
*Strombosia philippinensis* (Baill.) Rolfe	N/E	LC	NE	B
** Orchidaceae **				
Acriopsis liliifolia var. liliifolia (J.Koenig) Ormerod*	N/E	NE	NE	S
*Agrostophyllum loheri* Ormerod	E	NE	NE	M
*Appendicula laxifolia* J.J.Sm.*	N/E	NE	NE	S
*Appendicula leytensis* Ames	E	NE	NE	M
Appendicula reflexa var. reflexa Blume*	N/E	NE	NE	S
*Brachypeza unguiculata* (Lindl.) Kocyan & Schuit.	N/E	NE	NE	S
*Bulbophyllum leytense* Ames	E	NE	NE	M
*Bulbophyllum loherianum* (Kraenzl.) Ames	E	NE	EN	M
*Bulbophyllum longiflorum* Thouars	N/E	NE	NE	E
*Bulbophyllum maquilingense* Ames & Quisumb.	E	NE	NE	M
Bulbophyllum mucronatum subsp. alagense (Ames) J.J.Verm. & P.O'Byrne	N/E	NE	NE	M
*Calanthe furcata* Bateman ex Lindl.	E	NE	NE	S
*Calanthe triplicata* (Willemet) Ames	N/E	NE	NE	S
*Coelogyne asperata* Lindl.	N/E	NE	NE	E
*Coelogyne convallariiformis* (Schauer) M.W.Chase & Schuit.	E	NE	NE	M
*Coelogyne filiformis* (Lindl.) M.W.Chase & Schuit.	E	NE	NE	M
*Coelogyne sinuata* M.W.Chase & Schuit.	E	NE	NE	E
*Coelogyne tenella* (Nees & Meyen) M.W.Chase & Schuit.	E	NE	NE	M
*Corybas kaiganganianus* Tandang, A.S.Rob. & M.D.Angeles	E	CR^@^	CR	P
*Corymborkis veratrifolia* (Reinw.) Blume	N/E	NE	NE	S
*Crepidium bancanoides* (Ames) Szlach.*	E	NE	NE	S
*Crepidium binabayense* (Ames) Szlach.	E	NE	NE	M
*Crepidium moluccanum* (J.J.Sm.) Ormerod*	N/E	NE	NE	S
*Cryptostylis arachnites* (Blume) Hassk.	N/E	NE	NE	E
*Cymbidium aliciae* Quisumb.	N/E	NE	EN	M
*Cymboglossum cymbidiifolium* (Ridl.) Ormerod & Cootes*	N/E	NE	NE	S
*Dendrobium crumenatum* Sw.	N/E	NE	NE	S
*Dendrobium gerlandianum* Kraenzl.	E	NE	NE	M
*Dendrobium microphyton* L.O.Williams	N/E	NE	NE	M
*Dendrobium orbilobulatum* Fessel & Lückel	E	NE	NE	M
*Dendrobium uniflorum* Griff.	N/E	NE	NE	E
*Grammatophyllum wallisii* Rchb.f.	E	NE	CR	M
*Nervilia* sp.	-	-	-	S
*Orchipedum shareeanniae* Tandang, Ordas & S.G.Zamudio	SE	DD^@^	NE	S, X
*Peristylus goodyeroides* (D.Don) Lindl.*	N/E	NE	NE	S
*Phaius stenocentron* Schltr.*	N/E	NE	NE	S
*Phaius tankervilleae* (Banks) Blume	N/E	NE	NE	S
*Phalaenopsis deliciosa* Rchb.f.*	N/E	NE	NE	S
*Phalaenopsis lueddemanniana* Rchb.f.	E	NE	EN	M
*Pinalia polyura* (Lindl.) Kuntze	E	NE	NE	M
*Plocoglottis copelandii* Ames	E	LC	NE	E
*Plocoglottis plicata* (Roxb.) Ormerod*	N/E	NE	NE	S
*Podochilus intricatus* Ames	E	NE	NE	M
*Pseuderia samarana* Z.D.Meneses & Cootes	E	CR^@^	EN	N, A
*Pteroceras philippinense* (Ames) Garay	E	NE	NE	S, M
*Renanthera matutina* Lindl.	N/E	NE	VU	M
*Robiquetia compressa* (Lindl.) Schltr.*	E	NE	NE	S
*Robiquetia minimiflora* (Hook.f.) Kocyan & Schuit.	N/E	NE	NE	M
*Spathoglottis plicata* Blume	N/E	NE	NE	S, E
*Taeniophyllum philippinense* Rchb.f.*	E	NE	NE	S
*Thrixspermum elongatum* Ames	E	NE	NE	S
*Trichoglottis celebica* Rolfe	E	NE	NE	S
*Trichoglottis latisepala* Ames	E	NE	NE	S
*Trichoglottis loheriana* (Kraenzl.) L.O.Williams	E	NE	EN	M
*Vanilla ovalis* Blanco	E	NE	NE	M
*Vanilla raabii* Ormerod & Cootes	E	NE	NE	S
*Vrydagzynea albida* (Blume) Blume	N/E	NE	NE	E
** Pandanaceae **				
*Benstonea copelandii* (Merr.) Callm. & Buerki	E	LC	NE	C
*Freycinetia cumingiana* Gaudich.	E	NE	NE	S
*Freycinetia merrillii* Elmer*	E	NE	NE	S
*Freycinetia multiflora* Merr.	N/E	NE	NE	S
*Freycinetia oblongifolia* Merr.	E	NE	NE	E
*Freycinetia philippinensis* Hemsl.	E	NE	NE	S
*Freycinetia robinsonii* Merr.*	N/E	LC	NE	S
*Freycinetia vidalii* Hemsl.	N/E	NE	NE	E
*Pandanus biliranensis* Merr.	E	EN	NE	E
*Pandanus gracilis* Blanco*	E	NT	NE	S
*Pandanus multibracteatus* Merr.*	E	CR	NE	S
** Passifloraceae **				
*Passiflora foetida* L.	N/E	NE	NE	E
** Pentaphragmataceae **				
*Pentaphragma grandiflorum* Kurz	N/E	NE	OTS	E
** Phyllanthaceae **				
*Antidesma macgregorii* C.B.Rob.	E	LC	NE	E
*Antidesma tomentosum* Blume	N/E	LC	NE	S, E
*Aporosa banahaensis* (Elmer) Merr.	N/E	NT	NE	E
*Aporosa sphaeridiophora* Merr.	N/E	NT	NE	S
*Breynia androgyna* (L.) Chakrab. & N.P.Balakr.	N/E	LC	NE	S
*Breynia cernua* (Poir.) Müll.Arg.	N/E	LC	NE	E
*Breynia racemosa* (Blume) Müll.Arg.	N/E	LC	NE	E
*Bridelia glauca* Blume	N/E	LC	NE	A, C
*Cleistanthus everettii* C.B.Rob.	N/E	EN	NE	E
*Cleistanthus pedicellatus* Hook.f.	N/E	LC	NE	E
*Flueggea flexuosa* Müll.Arg.	N/E	LC	NE	S, B
*Glochidion cauliflorum* Merr.	E	NE	NE	E
*Glochidion triandrum* (Blanco) C.B.Rob.	E	LC	NE	B
*Phyllanthus caudatifolius* Merr.	E	NE	NE	S
*Phyllanthus lancifolius* Merr.	N/E	NE	NE	S
** Piperaceae **				
*Peperomia* sp. 1	-	-	-	S
*Peperomia* sp. 2	-	-	-	S
*Piper davaoense* C.DC.*	E	NE	NE	S
*Piper* sp. 1	-	-	-	S
*Piper* sp. 2	-	-	-	S
*Piper lanatum* Roxb.	N/E	NE	NE	S, E
*Piper macropiper* Pennant	N/E	NE	NE	E
*Piper myrmecophilum* C.DC.	E	NE	NE	E
*Piper philippinum* Miq.	N/E	NE	NE	E
** Poaceae **				
*Centotheca lappacea* (L.) Desv.	N/E	NE	NE	E
*Coix lacryma-jobi* L.	N/E	NE	NE	E
*Dinochloa luconiae* (Munro) Merr.	N/E	NE	NE	S, E
*Oplismenus compositus* (L.) P.Beauv.	N/E	LC	NE	E
*Paspalum conjugatum* P.J.Bergius	N/E	NE	NE	S
*Paspalum scrobiculatum* L.	N/E	LC	NE	E
*Schizostachyum lima* (Blanco) Merr.	N/E	NE	NE	E
*Sorghum propinquum* (Kunth) Hitchc.	N/E	NE	NE	E
** Podocarpaceae **				
*Podocarpus rumphii* Blume	N/E	NT	OTS	B
** Polygalaceae **				
*Polygala venenosa* Juss. ex Poir.	N/E	NE	NE	E
** Polypodiaceae **				
*Drynaria aglaomorpha* Christenh.	N/E	NE	VU	G
*Drynaria quercifolia* (L.) J.Sm.	N/E	NE	NE	S
*Goniophlebium persicifolium* Bedd.	N/E	NE	NE	S
*Lepisorus accedens* (Blume) Hosok.	N/E	NE	NE	E
*Lepisorus longifolius* (Blume) Holttum	N/E	NE	NE	S, G
*Lepisorus mucronatus* (Fée) Li Wang	N/E	NE	NE	G, E
*Leptochilus hemionitideus* (Wall. ex Mett.) Noot.	N/E	NE	NE	G
*Leptochilus insignis* (Blume) Fraser-Jenk.*	N/E	NE	NE	S
*Leptochilus macrophyllus* (Blume) Noot.	N/E	NE	NE	S, G, A
*Leptochilus pteropus* (Blume) Fraser-Jenk.	N/E	LC	NE	G, E
*Loxogramme avenia* (Blume) C.Presl	N/E	NE	NE	S
*Microsorum longissimum* Fée	N/E	NE	NE	G, E
*Microsorum membranifolium* (R.Br.) Ching	N/E	NE	NE	S, G
*Microsorum monstrosum* (Copel.) Copel.	E	NE	NE	S
*Microsorum punctatum* (L.) Copel.	N/E	NE	NE	S, G, E
*Microsorum samarense* (J.Sm.) Bosman	E	NE	NE	G
*Microsorum scolopendrium* (Burm.fil.) Copel.	N/E	NE	NE	S
*Oreogrammitis jagoriana* (Mett. ex Kuhn) Parris & Sundue	N/E	NE	NE	G
*Platycerium coronarium* (D.Koenig ex O.F.Müll.) Desv.	N/E	NE	CR	G, E
*Prosaptia cryptocarpa* Copel.	E	NE	NE	G, E
*Pyrrosia lanceolata* (L.) Farw.	N/E	NE	NE	S, G
*Pyrrosia longifolia* (Burm.f.) C.V.Morton	N/E	NE	NE	S
*Pyrrosia samarensis* (C.Presl) Ching	E	NE	NE	S, G
*Pyrrosia splendens* (C.Presl) Ching	E	NE	VU	S, D, G
*Selliguea lateritia* (Baker) Hovenkamp	N/E	NE	NE	G, E
*Selliguea taeniata* (Sw.) Parris	N/E	NE	NE	G
*Thylacopteris papillosa* Kze.; J.Sm.	N/E	NE	NE	G
** Primulaceae **				
*Ardisia elliptica* Thunb.	N/E	LC	VU	S, E
*Ardisia pyramidalis* (Cav.) Pers.	N/E	NE	NE	B
*Ardisia serrata* (Cav.) Pers.	N/E	LC	NE	E
*Ardisia warburgiana* Mez	E	NE	NE	S, E
*Discocalyx linearifolia* Elmer	E	NT	NE	E
*Embelia whitfordii* Merr.	E	NE	NE	E
** Pteridaceae **				
*Adiantum caudatum* L.*	N/E	NE	NE	S
*Adiantum diaphanum* Blume	N/E	NE	NE	G, E
*Antrophyum reticulatum* (G.Forst.) Kaulf.	N/E	NE	NE	G
*Antrophyum sessilifolium* (Cav.) Spreng.	N/E	NE	NE	S
*Austrogramme luzonica* (Alderw.) M.Kato	E	NE	NE	E
*Haplopteris alternans* (Copel.) S.Linds. & C.W.Chen	N/E	NE	NE	E
*Haplopteris mediosora* (Hayata) X.C.Zhang	N/E	NE	NE	G
*Pityrogramma calomelanos* (L.) Link	N/E	NE	NE	S
*Pteris ensiformis* Bunn.	N/E	NE	NE	A
*Pteris opaca* J.Sm. ex Hook.	N/E	NE	OTS	G
*Pteris tripartita* Sw.	N/E	NE	NE	S
*Pteris vittata* L.	N/E	LC	NE	S
*Taenitis blechnoides* (Willd.) Sw.	N/E	NE	NE	S
*Taenitis cordata* (Gaudich.) Holttum	N/E	NE	VU	G
*Vaginularia trichoidea* Fée	N/E	NE	NE	G, E
** Pteridryaceae **				
*Pteridrys microthecia* (Fée) C.Chr. & Ching	E	NE	NE	G
** Putranjivaceae **				
*Drypetes rhakodiskos* (Hassk.) Bakh.f.	N/E	LC	OTS	E
** Rhamnaceae **				
*Alphitonia philippinensis* Braid	N/E	LC	NE	B
** Rhizophoraceae **				
*Carallia brachiata* (Lour.) Merr.	N/E	LC	NE	B
*Pellacalyx pustulata* Merr.	N/E	NE	NE	E
** Rosaceae **				
*Prunus grisea* (Blume ex Müll.Berol.) Kalkman	N/E	LC	NE	E
*Prunus marsupialis* Kalkman	E	LC	NE	E
*Rubus moluccanus* L.	N/E	NE	NE	S, E
Rubus moluccanus var. discolor (Blume) Kalkman	N/E	NE	NE	S
** Rubiaceae **				
*Aidia acuminata* (Blume) K.M.Wong	N/E	NE	NE	E
*Antherostele grandistipula* (Merr.) Bremek.	E	VU	NE	S, E
*Argostemma maquilingense* Elmer	E	NE	NE	K
*Benkara microcarpa* (Bartl. ex DC.) Ridsdale	E	NE	NE	K
*Canthium* sp.	-	-	-	S
*Chewlunia* sp.	-	-	-	S
*Coptosapelta olaciformis* (Merr.) Elmer	E	NE	NE	E
*Diplospora sorsogonensis* (Elmer) A.P.Davis*	E	CR	NE	S
*Diplospora tinagoensis* (Elmer) S.J.Ali & Robbr.	N/E	NE	NE	E
*Dolicholobium philippinense* Trel.	E	NT	NE	K
*Eumachia membranifolia* (Bartl. ex DC.) Barrabé, C.M.Taylor & Razafim.	N/E	LC	NE	S
*Exallage auricularia* (L.) Bremek.	N/E	NE	NE	K
*Exallage buruensis* (Miq.) Bremek.	N/E	NE	NE	K
*Exallage cristata* (Willd.) Nandikar & K.C.Kishor	N/E	NE	NE	K
*Geophila repens* (L.) I.M. Johnst.	N/E	NE	NE	S
*Greeniopsis megalantha* Merr.	E	VU	NE	S
*Greeniopsis multiflora* (Elmer) Merr.	E	LC	NE	S, B, E
*Guettardella* sp.	-	-	-	S
*Gynochthodes platyphylla* (Merr.) Razafim. & B.Bremer	E	NE	NE	S
*Hedyotis longipedunculata* Merr.	E	NE	NE	K
*Hedyotis philippensis* (Willd. ex Spreng.) Merr. ex C.B.Rob.	N/E	NE	NE	S
*Hydnophytum formicarum* Jack	N/E	LC^@^	OTS	K, E
*Ixora bartlingii* Elmer	N/E	NE	NE	S, E
*Ixora cumingiana* S.Vidal	E	NE	NE	E
*Ixora filipes* Valeton	N/E	NE	NE	E
*Ixora leytensis* Elmer	E	NE	NE	S, E
*Ixora longifolia* Sm.	N/E	NE	NE	S
*Ixora macrophylla* Bartl. ex DC.	E	NE	NE	S
*Ixora salicifolia* (Blume) DC.	N/E	NE	NE	K
*Ixora silagoensis* Manalastas, Banag & Alejandro	E	CR^@^	NE	S, K
*Lasianthus attenuatus* Jack	N/E	NE	NE	S, K
*Lasianthus cyanocarpus* Jack*	N/E	NE	NE	S
*Lasianthus hirsutus* (Roxb.) Merr.	N/E	NE	NE	S, E, K
*Lasianthus* sp.	-	-	-	S
*Lasianthus trichophlebus* Hemsl.	N/E	NE	NE	C
*Lasianthus verticillatus* (Lour.) Merr.	N/E	NE	NE	K, E
*Leptopetalum biflorum* (L.) Neupane & N.Wikstr.	N/E	NE	NE	S
*Ludekia bernardoi* (Merr.) Ridsdale	E	NE	NE	E
*Ludekia* sp.	-	-	-	S
*Morinda citrifolia* L.	N/E	LC	NE	S
Morinda citrifolia var. bracteata (Roxb.) Kurz*	N/E	NE	NE	S
*Mussaenda philippica* A.Rich.	E	LC	NE	S
*Mussaenda vidalii* Elmer	E	NT	VU	K
*Mycetia javanica* (Blume) Reinw. ex Korth.	N/E	NE	NE	S, E
*Mycetia suedixieana* Tandang & Ordas	E	DD^@^	VU	T
*Myrmecodia tuberosa* Jack	N/E	NE	NE	K
Neonauclea bartlingii var. cumingiana (S.Vidal) Ridsdale	E	NE	NE	K
*Neonauclea formicaria* (Elmer) Merr.	E	LC	NE	B, C
*Neonauclea jagori* (Merr.) Merr.	E	EN	NE	S, K
*Neonauclea puberula* (Merr.) Merr.	E	VU	NE	B
*Neonauclea viridiflora* Ordas, Banag & Alejandro	SE	EN^@^	EN	S, K
*Neonauclea wenzelii* (Merr.) Merr.	E	EN	NE	K
*Ophiorrhiza acuminata* DC.	E	NE	NE	S
*Ophiorrhiza camiguinensis* Elmer	E	NE	NE	S, K
*Ophiorrhiza ciliata* Elmer*	E	NE	NE	S
*Ophiorrhiza* sp. 1	-	-	-	S
*Ophiorrhiza* sp. 2	-	-	-	S
*Ophiorrhiza* sp. 3	-	-	-	S
*Ophiorrhiza lancilimba* Merr.*	E	NE	NE	S
*Ophiorrhiza mungos* L.*	N/E	NE	NE	S
*Ophiorrhiza pubescens* Elmer	E	NE	NE	S
*Ophiorrhiza sorsogonensis* Elmer*	E	NE	NE	S
*Praravinia triflora* (Quisumb. & Merr.) Bremek.	E	NE	NE	K
Prismatomeris tetrandra subsp. tetrandra (Roxb.) K.Schum.	N/E	LC	NE	D, A
*Psychotria banahaensis* Elmer	E	LC	NE	E
*Psychotria conglomeratiflora* Sohmer & A.P.Davis	E	CR or possibly extict^@^	NE	K
*Psychotria crassifolia* Miq.*	N/E	LC	NE	S
*Psychotria nitens* (Merr.) Merr.	E	NE	NE	E
*Psychotria paloensis* Elmer	E	NT	NE	S
*Psychotria papillata* (Merr.) Merr.	E	VU^@^	NE	K
*Psychotria pilosella* Elmer	E	LC	NE	S, E
Psychotria pilosella var. pilosella Elmer	E	NT^@^	NE	S
*Psychotria radicans* (Merr.) Merr.	E	VU^@^	NE	K
Psychotria subsessiliflora var. subsessiliflora Elmer	E	NE	NE	E
Psychotria tayabensis var. fasciculiflora (Merr.) Sohmer & A.P.Davis	SE	NE	NE	E
*Psychotria yatesii* (Merr.) Merr.	E	VU	NE	E
*Psydrax amplifolius* (Elmer) A.P.Davis	E	NE	NE	K
*Scleromitrion verticillatum* (L.) R.J.Wang*	N/E	NE	NE	S
*Spermacoce remota* Lam.	N/E	LC	NE	K
*Tarenna cumingiana* (S.Vidal) Elmer	N/E	NE	NE	S
*Tarenna luzoniensis* (S.Vidal) Bremek.	E	NE	NE	K
*Timonius appendiculatus* Merr.	E	VU	NE	B
*Timonius confertiflorus* Merr.	E	NE	NE	E
*Timonius sulitii* Merr. & Quisumb. ex J.G.Chavez & Tandang	SE	CR^@^	CR	L
*Uncaria attenuata* Korth.	N/E	NE	NE	S, K
*Uncaria longiflora* (Poir.) Merr.	N/E	NE	NE	K
*Uncaria perrottetii* (A.Rich.) Merr.*	N/E	NE	NE	S
*Uncaria roxburghiana* Korth.	N/E	NE	NE	S, E
*Urophyllum leytense* Merr.	E	NE	NE	B
*Urophyllum memecyloides* (C.Presl) S.Vidal	E	NE	NE	S
*Urophyllum platyphyllum* Elmer*	E	NE	NE	S
*Urophyllum urdanetense* Elmer	E	NE	NE	K
*Urophyllum* sp.	-	-	-	S
*Villaria purpurea* (Elmer) Arriola & Alejandro	E	NE	NE	E
*Xanthophytum ferrugineum* (DC.) Merr.	N/E	NE	NE	S, E
** Rutaceae **				
Glycosmis cyanocarpa var. platyphylla (Merr.) B.C.Stone	N/E	LC	NE	S
*Lunasia amara* Blanco	N/E	LC	NE	E
Lunasia amara var. amara Blanco	N/E	LC	NE	S
*Melicope lunu-ankenda* (Gaertn.) T.G.Hartley	N/E	LC	OTS	B
*Melicope sessilifoliola* (Merr.) T.G.Hartley	E	EN	NE	B
*Melicope triphylla* (Lam.) Merr.	N/E	LC	NE	S, E
*Wenzelia brevipes* Merr.	E	NE	NE	E
*Zanthoxylum integrifoliolum* (Merr.) Merr.	N/E	LC	NE	E
Zanthoxylum myriacanthum var. myriacanthum Wall. ex Hook.f.	N/E	LC	NE	B
** Salicaceae **				
*Casearia euphlebia* Merr.	E	NE	NE	S
*Casearia* sp.	-	-	-	S
*Casearia phanerophlebia* Merr.*	E	VU	NE	S
*Homalium samarense* Merr.	E	VU	NE	B
*Osmelia philippina* (Turcz.) Fern.-Vill.	N/E	LC	NE	E
** Sapindaceae **				
Dictyoneura acuminata subsp. acuminata Blume	N/E	LC	NE	S
*Gloeocarpus patentivalvis* (Radlk.) Radlk.	E	NT	EN	A
*Guioa discolor* Radlk.	E	VU	OTS	A
*Guioa koelreuteria* (Blanco) Merr.	N/E	LC	NE	F
*Harpullia cupanioides* Roxb.*	N/E	LC	NE	S
*Lepisanthes senegalensis* (Juss. ex Poir.) Leenh.	N/E	LC	NE	E
*Lepisanthes tetraphylla* (Vahl) Radlk.	N/E	LC	NE	F
*Nephelium ramboutan-ake* (Labill.) Leenh.	N/E	LC	VU	F, B
*Pometia pinnata* J.R.Forst. & G.Forst.	N/E	LC	NE	S
*Rhysotoechia ramiflora* Radlk.	N/E	NE	NE	E
** Sapotaceae **				
*Abebaia fasciculata* (Warb.) Baehni	N/E	VU	NE	F, A, C, E
*Mimusops elengi* L.	N/E	LC	NE	B
*Palaquium elongatum* Merr.	E	EN	NE	A, C, E
*Palaquium luzoniense* (Fern.-Vill.) Vidal	N/E	LC	OTS	B
*Palaquium obovatum* (Griff.) Engl.	N/E	LC	NE	S
*Palaquium philippense* (Perr.) C.B.Rob.	E	LC	OTS	F
*Planchonella velutina* (Elmer) H.J.Lam	E	NT	NE	B, C
** Schizaeaceae **				
*Schizaea dichotoma* (L.) Sm.	N/E	NE	NE	G
** Selaginellaceae **				
*Selaginella alligans* Hieron.*	N/E	NE	NE	S
*Selaginella aristata* Spring	N/E	NE	NE	G
*Selaginella biformis* A.Braun ex Kuhn	N/E	NE	NE	S, G
*Selaginella ciliaris* (Retz.) Spring	N/E	NE	NE	G
*Selaginella cumingiana* Spring	N/E	NE	NE	G, E
*Selaginella cupressina* (Willd.) Spring	N/E	NE	NE	S, G, E
*Selaginella engleri* Hieron.	N/E	NE	NE	S, D, G, E
*Selaginella involvens* (Sw.) Spring	N/E	NE	NE	G
*Selaginella latifrons* Warb.	E	NE	NE	S
*Selaginella llanosii* Hieron.	N/E	NE	NE	E
*Selaginella luzonensis* Hieron.	E	NE	NE	E
** Stemonuraceae **				
*Gomphandra fernandoi* Schori & Utteridge	E	VU	VU	A
*Gomphandra mappioides* Valeton	N/E	LC	NE	S, D, A, E
*Medusanthera laxiflora* (Miers) R.A.Howard	N/E	LC	NE	E
** Symplocaceae **				
Symplocos odoratissima var. odoratissima (Blume) Choisy ex. Zoll.	N/E	LC	NE	F
** Tectariaceae **				
*Polydictyum menyanthidis* (C.Presl) C.Presl	N/E	NE	NE	G, E
*Tectaria angulata* (Willd.) Copel.	N/E	NE	NE	G
*Tectaria aspidioides* (C.Presl) Copel.	N/E	NE	NE	G, E
*Tectaria athyriosora* M.G. Price	E	NE	NE	G, A, E
*Tectaria aurita* (Sw.) S.Chandra	N/E	NE	NE	G, E
*Tectaria beccariana* (Ces.) C.Chr.	N/E	NE	NE	G, E
*Tectaria calcarea* (C. Presl) Copel.	E	NE	NE	A, G, E
*Tectaria crenata* Cav.	N/E	NE	NE	G, E
*Tectaria decurrens* (C.Presl) Copel.	N/E	NE	NE	S, G
*Tectaria dissecta* (G. Forst.) Lellinger	N/E	NE	NE	G, E, A
*Tectaria macleanii* (Copel.) S.Y.Dong	E	NE	EN	G, E
*Tectaria multicaudata* (C.B.Clarke) Ching	N/E	NE	NE	G, E
*Tectaria psomiocarpa* S.Y.Dong	E	NE	VU	S, G
*Tectaria ramosii* (Copel.) Holttum	E	NE	NE	G, E
*Tectaria samariana* S.Y.Dong	SE	NE	NE	G, E
*Tectaria trifida* (Fée) M.G.Price	E	NE	NE	G, E
** Theaceae **				
*Camellia lanceolata* (Blume) Seem.	N/E	LC	OTS	E
** Thelypteridaceae **				
*Chingia christii* (Copel.) Holttum*	E	NE	NE	S
*Grypothrix cuspidata* (Blume) S.E.Fawc. & A.R.Sm.	N/E	NE	NE	G
*Mesopteris attenuata* (Kuntze) S.E.Fawc. & A.R.Sm.	N/E	NE	NE	G, E
*Pneumatopteris glabra* (Copel.) Holttum	E	NE	NE	S
*Pneumatopteris lithophila* Holttum	E	NE	NE	G
*Pronephrium granulosum* (C.Presl) Holttum	N/E	NE	NE	G
*Pronephrium rhombeum* (Christ) Holttum	N/E	NE	NE	G, A
*Pronephrium xiphioides* (Christ) Holttum	E	NE	NE	S
*Reholttumia laevis* (Mett.) S.E.Fawc. & A.R.Sm.	E	NE	NE	G, E
*Reholttumia nitidula* (C.Presl) S.E.Fawc. & A.R.Sm.	E	NE	NE	G, E
*Sphaerostephanos convergens* Holttum	SE	NE	NE	G, E
*Sphaerostephanos hernaezii* Holttum	SE	NE	VU	G, E
*Sphaerostephanos productus* (Kaulf.) Holttum	N/E	NE	NE	G
*Sphaerostephanos subcordatus* Holttum	SE	NE	NE	G
Thymelaeaceae				
*Aquilaria cumingiana* (Decne.) Ridl.	N/E	VU	EN	F, C, S
*Gonystylus reticulatus* Merr.	E	EN	NE	A, C
*Phaleria capitata* Jack*	N/E	LC	NE	S
** Urticaceae **				
*Elatostema agusanense* Elmer	E	NE	NE	E
*Elatostema glaucescens* Wedd.*	E	NE	NE	S
*Elatostema integrifolium* (D.Don) Wedd.	N/E	NE	NE	E
*Elatostema* sp.	-	-	-	S
*Elatostema volubile* (Elmer) H.Schroet.	E	NE	NE	S
*Leucosyke capitellata (*Poir.) Wedd.	N/E	LC	NE	E
*Pipturus asper* Wedd.	N/E	LC	NE	S, E
*Poikilospermum suaveolens* (Blume) Merr.	N/E	NE	NE	S
*Pouzolzia zeylanica* (L.) Benn.	N/E	NE	NE	E
** Viscaceae **				
*Ginalloa arnottiana* Korth.	N/E	NE	NE	S
** Vitaceae **				
*Ampelocissus madulidii* Latiff	E	NE	NE	S
*Causonis pterita* (Merr.) J.Wen & L.M.Lu*	N/E	NE	NE	S
*Cayratia apoensis* (Elmer) Quisumb.	E	NE	NE	E
*Cayratia mollissima* (Wall.) Gagnep.	N/E	NE	NE	E
*Leea aculeata* Blume ex Spreng.	N/E	LC	NE	S
*Leea aequata* L.*	N/E	LC	NE	S
*Leea guineensis* G.Don	N/E	NE	NE	S, E
*Leea magnifolia* Merr.	E	NT	NE	E
*Leea manillensis* Walp.	N/E	NE	NE	E
*Leea quadrifida* Merr.	E	LC	NE	S
*Tetrastigma loheri* Gagnep.	N/E	NE	NE	S
*Tetrastigma papillosum* (Blume) Planch.*	N/E	NE	NE	S
** Zingiberaceae **				
*Adelmeria isarogensis* Docot & Banag*	E	EN^@^	EN	S
*Alpinia apoensis* Elmer*	E	VU^@^	NE	S
*Alpinia brevilabris* C.Presl	E	NE	NE	S, E
*Alpinia cumingii* K.Schum.	E	NE	NE	S
*Alpinia elegans* (C.Presl) K.Schum.	E	NE	NE	S, E
*Alpinia haenkei* C. Presl.	E	LC	NE	S, E
*Alpinia rufa* (C.Presl) Náves	E	NE	NE	S, E
*Etlingera cinnabarina* Docot & Ordas	SE	EN^@^	NE	S, W
*Etlingera coccinea* (Blume) S.Sakai & Nagam.*	N/E	LC	NE	S
*Etlingera fimbriobracteata* (K.Schum.) R.M.Sm.*	N/E	LC	NE	S
*Etlingera funakoshii* Docot	SE	CR^@^	NE	S, W
*Etlingera rigida* Docot	SE	CR^@^	NE	S, W
*Geocharis fusiformis* (Ridl.) R.M.Sm.	N/E	EN	NE	S
*Globba campsophylla* K.Schum.	E	LC	NE	S
*Globba parviflora* C.Presl	E	NE	NE	D
*Hedychium coronarium* Koenig*	N/E	DD	NE	S
*Hellwigia orientalis* (Docot & Banag) Senjaya & A.D.Poulsen*	E	EN^@^	NE	S
*Hornstedtia conoidea* Ridl. in Elmer*	E	NE	NE	S
*Meistera propinqua* (Ridl.) Škornick. & M.F.Newman	E	NE	NE	S, E
*Plagiostachys albiflora* Ridl.*	N/E	LC	NE	S
*Plagiostachys escritorii* Elmer	E	NE	NE	S
*Plagiostachys philippinensis* Ridl.	E	NE	NE	S
*Zingiber subroseum* Docot*	E	CR^@^	CR	S
*Zingiber zerumbet* (L.) Roscoe ex Sm.*	N/E	DD	NE	S
